# Promoter Nucleosome Organization Shapes the Evolution of Gene Expression

**DOI:** 10.1371/journal.pgen.1002579

**Published:** 2012-03-15

**Authors:** Dalia Rosin, Gil Hornung, Itay Tirosh, Ariel Gispan, Naama Barkai

**Affiliations:** Department of Molecular Genetics, Weizmann Institute of Science, Rehovot, Israel; University of California San Francisco, United States of America

## Abstract

Understanding why genes evolve at different rates is fundamental to evolutionary thinking. In species of the budding yeast, the rate at which genes diverge in expression correlates with the organization of their promoter nucleosomes: genes lacking a nucleosome-free region (denoted OPN for “Occupied Proximal Nucleosomes”) vary widely between the species, while the expression of those containing NFR (denoted DPN for “Depleted Proximal Nucleosomes”) remains largely conserved. To examine if early evolutionary dynamics contributes to this difference in divergence, we artificially selected for high expression of GFP–fused proteins. Surprisingly, selection was equally successful for OPN and DPN genes, with ∼80% of genes in each group stably increasing in expression by a similar amount. Notably, the two groups adapted by distinct mechanisms: DPN–selected strains duplicated large genomic regions, while OPN–selected strains favored *trans* mutations not involving duplications. When selection was removed, DPN (but not OPN) genes reverted rapidly to wild-type expression levels, consistent with their lower diversity between species. Our results suggest that promoter organization constrains the early evolutionary dynamics and in this way biases the path of long-term evolution.

## Author Summary

Species diverge by mutations that change protein structure or protein regulation. While the evolution of protein sequence was studied extensively, much less is known about the divergence of gene expression. To better understand the process of gene expression evolution, we characterized the early genomic response of yeast cells to selection for high gene expression. Notably, the response to selection was strongly dependent on the organization of the gene promoter: genes whose promoters had a pronounced nucleosome-free region (NFR) primarily duplicated the chromosome containing the gene of interest, while genes whose promoters lacked a pronounced NFR adapted by *trans* mutations not involving duplications. Further, when selection was removed, the former (but not the later) evolved strains reverted rapidly to wild-type expression levels, consistent with their lower diversity between species. Together, our study provides strong support to the idea that physiological regulation impacts the evolutionary path and suggests that, by regulating promoter nucleosomes, cells can regulate the response to selection and control the long-term stability of the selected changes.

## Introduction

The plasticity of biological traits is manifested on multiple time scales. Regulatory mechanisms govern the physiological adaptation of an individual to changing conditions. On evolutionary time scales, phenotypes are modulated by genetic mutations. Although operating on very different time scales, regulatory variance and evolvability were proposed to be linked [1]–[8]. For example, a trait that needs to be buffered against environmental or stochastic variations will show a limited regulatory variance, and will be harder to perturb by genetic mutations. A related idea is that regulatory variance directs the evolutionary dynamics by marking the directions most susceptible to changes. Experimental evidences supporting these ideas are still sparse, but recent studies in yeast provided genome-wide support to this linkage in the context of gene expression.

Adaptation of cells to different environmental conditions depends largely on changes in expression levels, whereas evolution depends on changes in both expression and function. While most studies of evolutionary changes focused on changes in gene function, the role of gene expression in generating phenotypic diversity was emphasized by experiments that traced phenotypic and morphological differences to variations in gene expression [9]–[12] and by genome-wide mapping of gene expression profiles which demonstrated rapid divergence even between closely related species [13]–[19].

In yeast, the divergence of gene expression was linked to the organization of promoter nucleosomes, thereby connecting evolutionary divergence with physiological regulation [20]–[22]. Genes whose expression diverged rapidly typically lack an NFR proximal to the transcription start site (OPN genes), while the expression of genes with a pronounced proximal NFR (DPN genes) remained largely conserved. OPN genes are additionally more responsive to environmental changes, display a higher cell-to-cell variability (noise) and tend to have a TATA box in their promoters [20].

Multiple, not mutually exclusive, processes can explain the increased divergence of OPN genes. First, the highly responsive OPN promoters may be more sensitive to mutations accumulating by random drift. This promoter organization may enhance sensitivity to *cis* mutation in the promoter itself due, for example to non-linear interactions between promoter nucleosomes and transcription factors. Similarly, the spectrum of effective *trans* mutations may be larger for OPN genes [22]. Indeed, OPN promoters integrate a larger number of signals, are more responsive to regulatory factors and diverge more in mutation-accumulation experiments where selection is eliminated [23].

A second possibility is the two classes of genes are subject to distinct selection forces. Increased expression of low-responsive (DPN) genes may be more deleterious and will therefore be eliminated more efficiently by purifying selection. Similarly, mutations in high-responsive genes may contribute more to evolutionary adaptation leading to their rapid fixation. Alternatively, DPN and OPN genes may be subject to similar selection forces for changing expression, but selection is more easily satisfied by OPN genes due to their flexible promoter structure. This last possibility would provide a direct support to the idea that flexible promoter organization can direct the dynamic path taken by evolution.

To examine the hypothesis that OPN and DPN genes differ in the way by which they respond to identical selection forces, we artificially selected for high expression of GFP-fused yeast proteins and examined the genomic response to this selection. The expression of dozens of GFP-fused proteins was successfully increased, irrespectively of their promoter class. Notably, promoter class did influence the genetic change leading to the increased expression: Selection for high expression of DPN genes resulted in duplication of large genomic region (mostly full chromosomes) containing the gene of interest. In contrast, large-scale duplications were much less prevalent in OPN genes, which changed their expression primarily through *trans* mutations not involving duplications. When selection was removed, DPN (but not OPN) strains reverted back to wild-type expression levels, consistent with their lower diversity between species. Our results suggest that promoter organization impacts on the early evolutionary dynamics and by this biases the path of long-term evolution.

## Results

**Figure 1:**
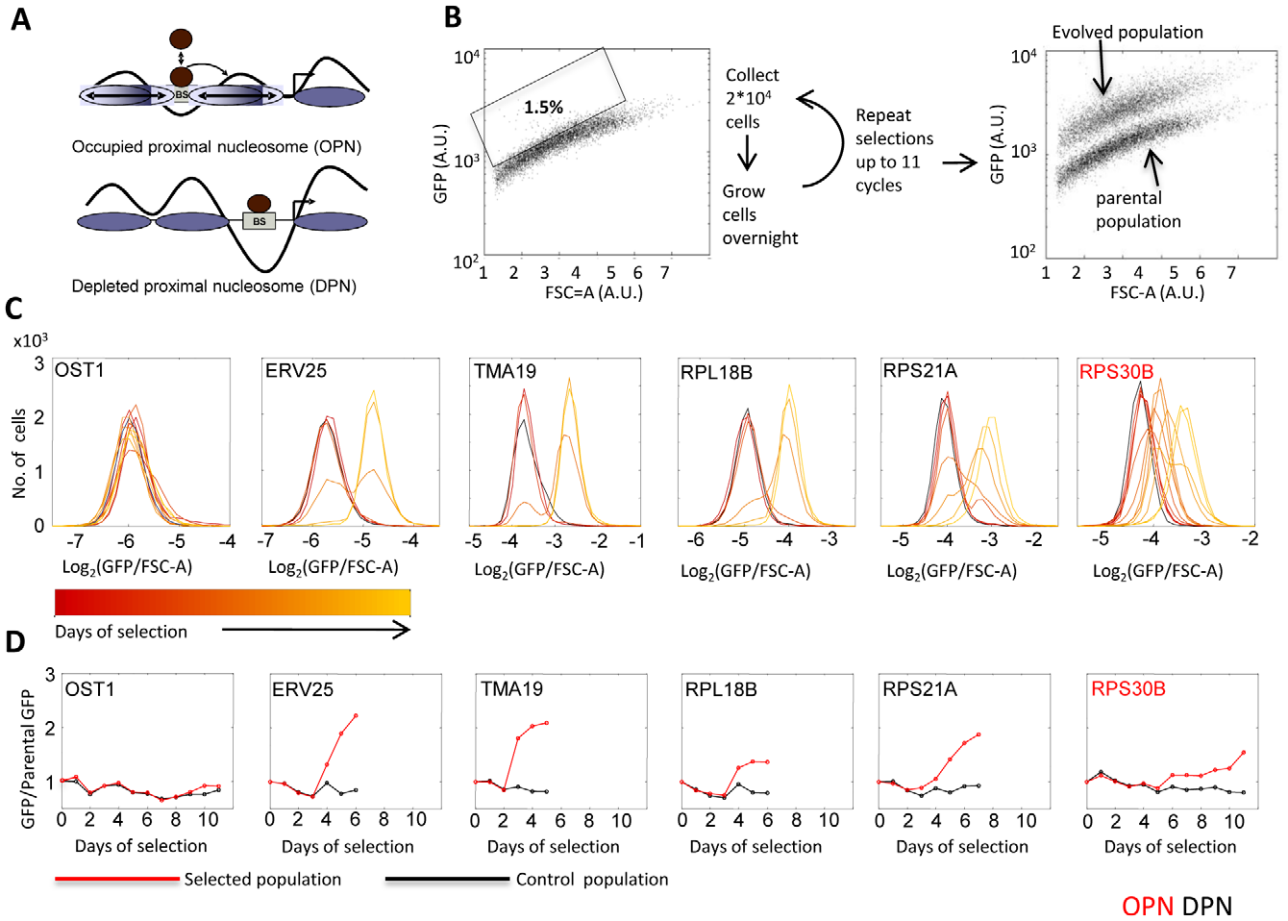
Selection for high gene expression. A: OPN versus DPN promoter organization: Schematic representation of nucleosome organization in promoters of the OPN and DPN classes. B. Selection for high expression: Strains expressing a GFP-fused protein were obtained. FACS was used to select the 1.5% cells with highest fluorescence, normalized by the forward scatter area (FSC-A). The distribution of FSC-A did not change between the parental and evolved strain, indicating that selection did not increase cell size (Materials and Methods). C–D: Increased expression in response to selection: The distributions (C) and medians (D) of fluorescence levels at successive days of selection are shown for representative genes. Fluorescence values are normalized to FSC-A (Materials and Methods). Data for all strains is given in Figure S2.

We chose forty-one yeast proteins that span a range of mid-to-high expression levels, with no preferences towards specific functions or positions along the chromosome (Table S1 and Figure S1). Genes were distributed between the DPN and OPN classes, as quantified by the relative nucleosome occupancy of their proximal promoter (‘OPN-measure’) [20]. The OPN-measure is defined as the ratio between nucleosome occupancy of the proximal versus distal region of the promoter, thereby quantifying the extent by which the proximal promoter region is depleted of nucleosomes. This measure strongly correlate with the flexibility of gene expression [20]. For each selected gene, we obtained the corresponding GFP-fusion protein [24] and used its fluorescence to select for high-expressing cells. Specifically, the distribution of fluorescence within the cell population was monitored using fluorescence activated cell sorter (FACS), and the top 1.5% (20,000) cells displaying the highest fluorescence levels (normalized by FSC-A which is an indicator of cell size) were collected (Figure 1B, Materials and Methods). The selected cells were grown, and the selection procedure was repeated until a clear shift in mean expression was observed, or up to a maximum of eleven cycles. No shift was observed in the FSC-A distribution, indicating that selection did not increase cell size.

Selection for high expression was successful in the majority of cases (35/41) (see Figure S3 caption for definition of success). Most genes increased expression after 3–6 rounds of selection. In some cases, expression increased gradually over subsequent cycles, perhaps reflecting the co-existence of multiple mutations with similar effects. Notably, expression increased by a typical 1.5 to 3 fold, and the extent of this change was not correlated with the promoter type (Figure 1C and 1D and Figures S2 and S3). The evolved high expression was stable for many generations. No significant change in mean expression was observed in control experiments in which the identical procedure was used but cells were FACS collected without selection.

To further understand the genetic mechanisms leading to increased expression, we first asked if the driving mutations are dominant or recessive. If the mutation causing the increased expression is dominant, high GFP expression will be maintained also when crossing the haploid evolved strain with a wild-type strain. In contrast, if the mutation is recessive, the level of GFP fluorescence will be reduced or even lost after crossing with a wild-type strain. Any mutation which is linked to the GFP locus (e.g. mutation in the promoter or gene duplication) will show a dominant effect in our assay. In contrast, mutations in *trans* regulators can be either dominant or recessive, depending on whether their impact is maintained or reduced when combined with the wild-type allele.

**Figure 2:**
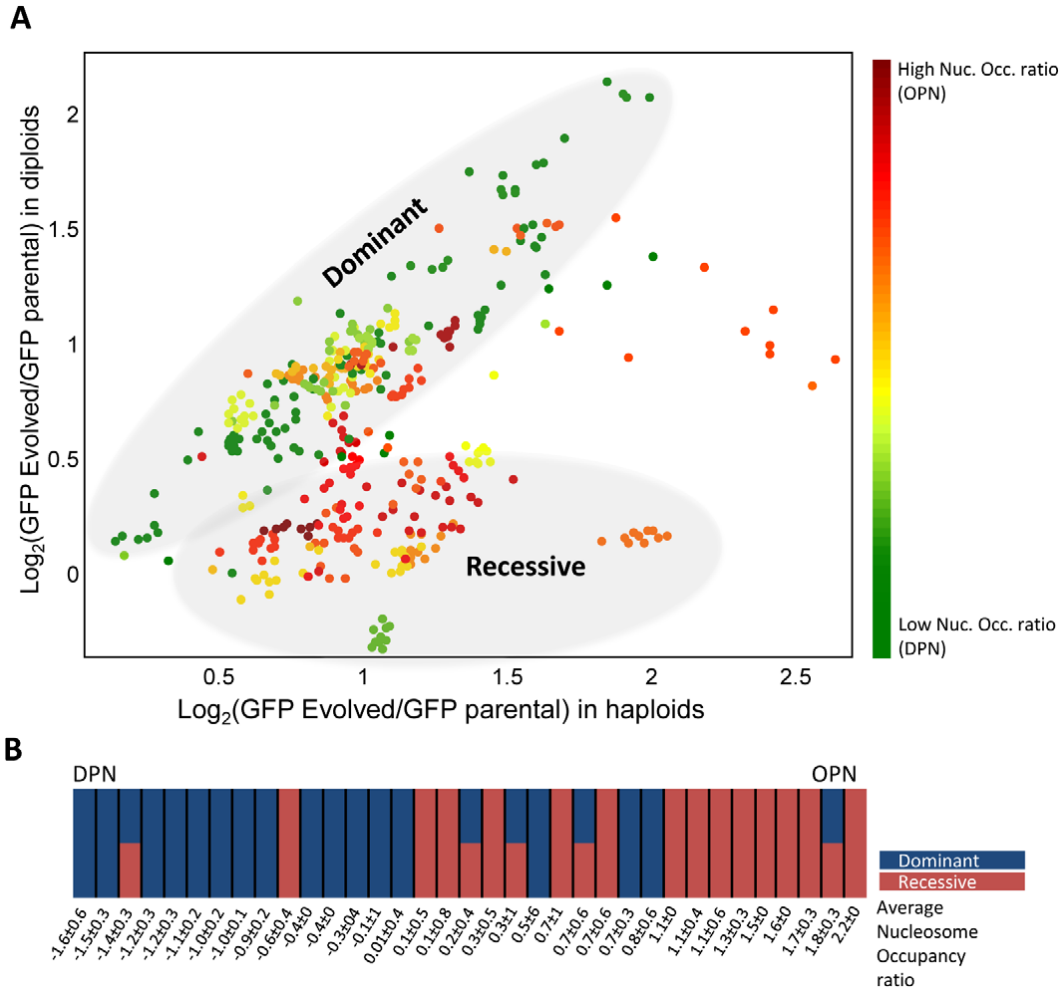
Genomic changes correlate with the gene promoter structure. A. Classifying mutations into dominant versus recessive: Eleven single colonies were isolated from each of the evolved strains and the underlying mutations were classified as dominant versus recessive by mating with a WT strain. The parental strain was mated with the same WT strain, and was used as a base-line for defining the fluorescence increase. Shown is the increase in fluorescence in the heterozygote diploids versus the increase in the respective haploids. The color code depicts the nucleosomes occupancy score (Materials and Methods). In five cases, a single gene was associated with both dominant and recessive colonies, as shown in B. For details of each of the individual strains see Figure S3. B. Dominant versus recessive mutations correlate with the DPN versus OPN promoter organizations: genes are organized according to the nucleosome occupancy score and are color-coded based on the mode of evolution (dominant versus recessive). The mean OPN measure (ratio of nucleosome occupancy at the promoter proximal versus distal region) of the dominant mutation is −0.5219 (with std of 0.7619) while that of the recessive mutations is 0.9129 (std of 0.7468) the two distributions differ with a p-value of p = 1.6364e-005. See Table S1 for further summary of the results.

Strikingly, genes of the DPN class evolved almost exclusively by dominant mutations, whereas OPN genes were mostly associated with recessive mutations that either eliminated or significantly reduced the expression of the evolved GFP allele in the diploid background (Figure 2 and Figure S3). Thus, of the fifteen DPN genes that evolved higher expression, 13 were classified as dominant (all single-colonies isolated from the evolved population maintained their high expression upon mating with a wild-type strain), one was recessive (all single-colonies showed reduced expression in a heterozygote background), and one population contained a mixture of dominant and recessive colonies, indicating two modes of evolution. In sharp contrast, of the 20 OPN genes, 13 were classified as recessive, four were a mixture of dominant and recessive colonies, and only three were dominant.

When directly comparing the nucleosome organization pattern (OPN score) of the genes evolving by dominant versus recessive mutations, we found the two distributions to be distinct with a p-value of 1.63*10^−5^. To further verify the reproducibility of the results, we repeated the selection procedure for 26 of the strains. Of the ten DPN genes evolved in this second round of validation only one gene changed its classification from dominant to a mixture of dominant and recessive. For the OPN genes, of the 16 genes examined in the second round twelve retained the same mode of evolution, one changed its classification from recessive to dominant, 2 changed from a mixture of dominant and recessive to dominant only and one did not evolve (Table S1). Combining the two experiments together, the hypothesis that the frequency of dominant versus recessive mutations depends on nucleosome organization is supported with a p-value of 1.26*10^−6^.

Next, we asked whether the mutations driving GFP expression change occurred in *cis* or in *trans*. *Cis* mutations are linked to the gene itself, and can be generated for example by mutations in the gene promoter or by gene duplication. As mentioned above, such mutations are necessarily dominant in our assay. In contrast, *trans* mutations may be either dominant or recessive. Mutations can be classified as *cis* or *trans* by examining the expression of the wild-type allele of the evolved gene within the evolved cells. Mutations in *cis* will have no effect on this second (wild-type) copy, while *trans* mutations are expected to increase also the expression of the second copy.

We therefore generated heterozygote diploids by mating the evolved colonies with wild-type cells in which the allele corresponding to the evolved gene was fused to mCherry. Notably, all the dominant mutations were classified as *cis*, showing no increase in mCherry expression (Figure S4). In contrast, coordinate elevation of GFP and mCherry levels were observed in all recessive cases where the evolved expression levels were only partially compensated in the diploid background. Taken together, we conclude that DPNs evolved primarily by dominant *cis* mutations while OPNs typically evolved by recessive *trans* mutations. We observed no correlation between the mode of evolution and the initial expression level, the presence of a TATA box [23] or repeats in the promoter sequence [25], initial noise or chromosomal position (Table S1).

**Figure 3:**
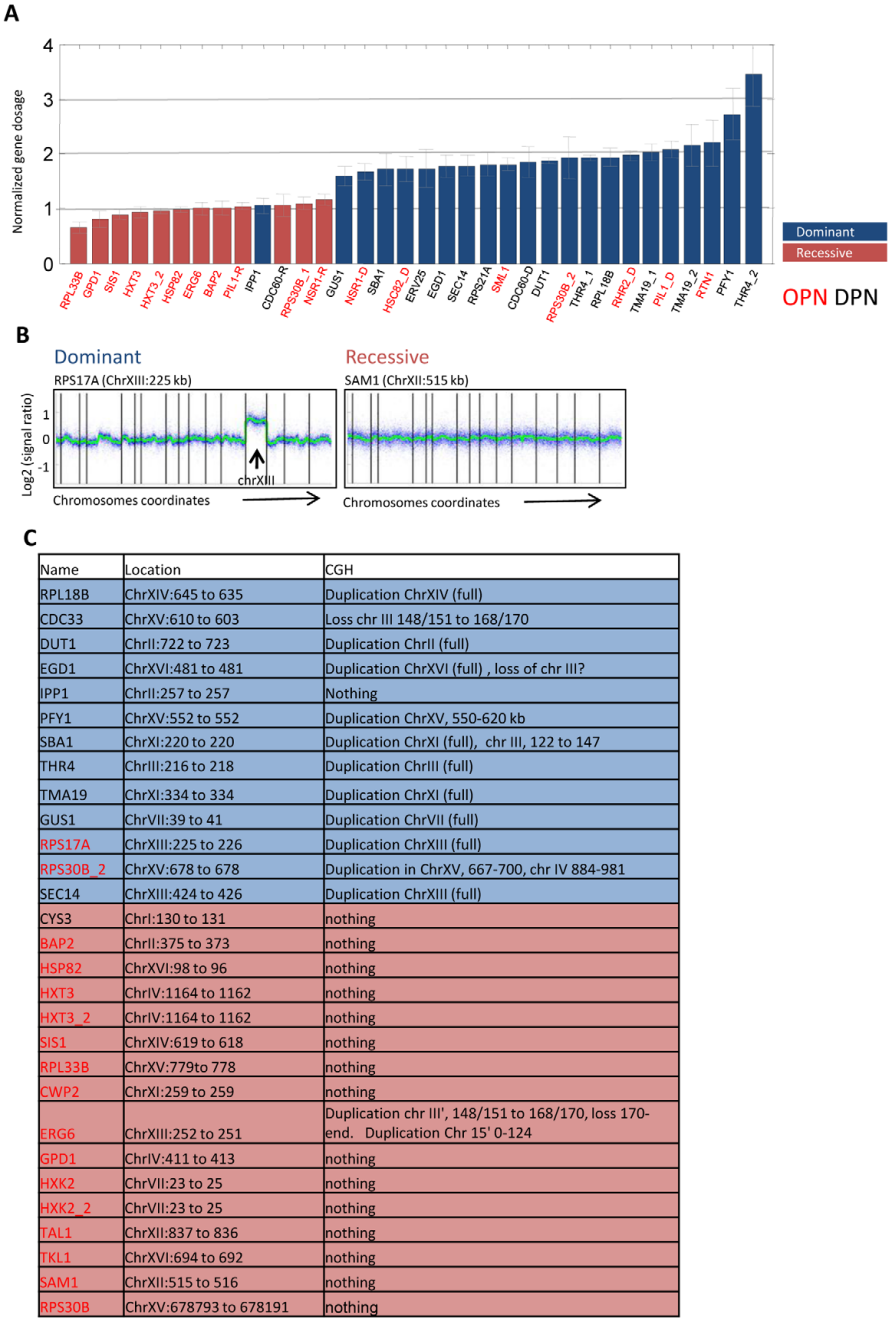
Dominant mutations involve large-scale genomic duplications. A. GFP copy number in the evolved strains: GFP DNA copy number was measured using real-time PCR. Values shown are normalized and are averaged over two biological repeats of three single colonies from each evolved strain. Two genes (RPS17A and CDC33) are omitted since we could not obtain reproducible results. These genes were analyzed for duplications by CGH. Dominant and recessive colonies associated with the same gene were analyzed separately and are shown with the corresponding D and R labels. Cases of repeated evolution are also shown (e.g. HXT3_1 and HXT3_2). The color code distinguish dominant versus recessive mutations. B–C. Dominant mutations involve large-scale genomic duplications: CGH analysis was used to define genomic rearrangements in the evolved strains. Shown are the hybridization ratios (relative to wt strain) of probes ordered by their genomic location. An example of a dominant strain harboring full chromosome duplication and a recessive strain with no apparent genomic rearrangements are shown. Vertical bars mark chromosome ends. Results for all strains are summarized in Table S1 (C, see also Figure S5). Color code is as in A.

Many of the dominant mutations increased expression by about two fold. To examine whether they present duplication of the associated gene, we measured the GFP DNA copy-number using real time PCR. With the exception of two cases, dominant mutations all involved gene duplication (Figure 3A). To define the duplicated region, we used an array-based comparative-genomic hybridization (CGH). Notably, large-scale duplications were identified in 11/13 dominant cases we assayed. Typically full chromosomes were duplicated (9/11), and the duplications invariably spanned the gene subject to selection (Figure 3B and 3C, and Figure S5). In principle, *trans* mutations could also result from duplications of regulatory genes. Yet, in only one of the sixteen recessive cases we examined we observed a duplication of a small chromosome fragment.

**Figure 4:**
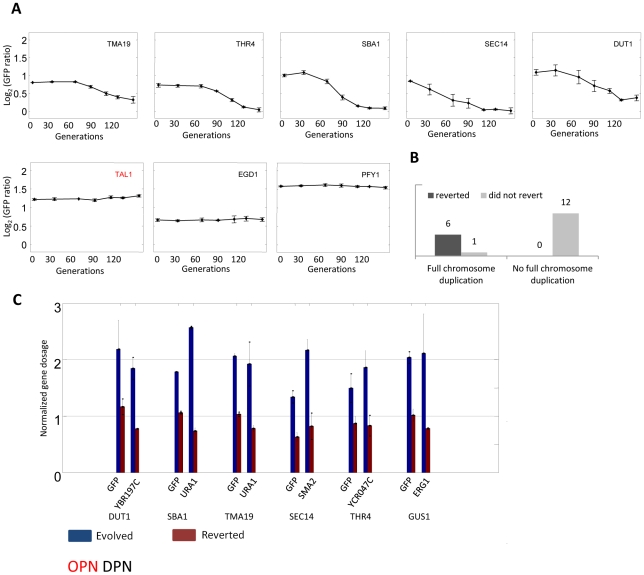
Reversion of evolution by chromosome duplication. Nineteen of the evolved strains were repeatedly diluted for ∼130 generations in SC. A. The temporal change in GFP fluorescence levels (normalized to its value in the parental strain) is shown for representative strains (see also Figure S6). B. Summarizes the results for all tested strains. C. Reversion of the evolved phenotype is due to loss of chromosome duplication. For each strain GFP copy number as well as copy number of independent locus of the relevant chromosome were measured by real time PCR.

We measured the competitive fitness of the evolved strains. The majority of strains displayed some growth defect, and there was no apparent distinction between the recessive and the dominant mutations (Figure S7). Still, since missegregation of chromosomes during cell division is a relatively common event [26], [27], we hypothesized that strains evolving by large scale duplications will revert faster than these evolving by other means once selection for GFP expression is removed. To examine that, we grew nineteen of the evolved strains for ∼130 generations and monitored GFP levels temporally. Out of seven strains with duplicated chromosomes tested, six reverted to their pre-selected expression level and this reversion was caused by the loss of the duplicated chromosome (Figure 4). In contrast, all twelve strains without such duplication maintained the evolved high expression (Figure 4 and Figure S6). It is likely that those strains improved their growth rate through alternative, compensating, mutations and not by reverting the original mutation leading to the increase GFP expression. Together, our results suggest that although both DPN and OPN gene groups evolve initially at the same rate, the solutions found by DPN genes is less likely to be maintained in the long term. This effectively results in DPN strains having fewer evolutionary solutions available for increasing gene expression in evolutionary time scale.

## Discussion

Genetic changes leading to increased gene expression can be classified as regulatory *cis*-effects, regulatory *trans*-effects and segmental duplications that increase gene's copy number. By regulatory *cis*-effects we refer to mutations in the close vicinity of the gene promoters, altering, for example, the binding of regulatory factors. *Trans*-effects refer to mutations that occur elsewhere in the genome, for example modulating the activity of an upstream transcription factor or signaling protein. Such mutations are expected to have a wider influence on gene expression compared to *cis*-effects as they will modulate the expression of many (or all) targets of the associated *trans* factor. Finally, gene expression can also be increased by duplication, consisting of either a full chromosome or a chromosomal region containing the gene of interest. Chromosome duplications will modulate the expression of hundreds of unrelated genes.

Studies that compared gene expression between related organisms revealed that most expression changes result from regulatory *cis* and *trans* mutations, with *cis*-effects dominating the divergence between species, while *trans*-effects dominate the divergence between different strains of the same species [18], [28], [29]. At least in yeast, *trans*-effects preferentially modulate the expression of genes of the OPN promoter type, but are less significant at genes of the DPN class [18]. Large scale chromosomal duplications are typically not observed when comparing yeast strains and species. This distribution of effects could reflect the frequency of initial mutations arising in the population, the interplay between their selective advantages versus possible deleterious outcomes, or the probability of reverting back the original mutations.

Our results provide a complementary view on the early response to selection for high expression. We find that the genetic changes dominating this initial adaptation differ from those dominating the long-term evolution. First, regulatory *cis* effects were not observed. Rather, expression was modulated either by large-scale duplications or by regulatory *trans* mutations. Most notably, the choice between *trans* mutations and large-scale duplications was essentially dictated by the gene promoter class: genes of the DPN class changed expression almost exclusively through duplications, whereas genes of the OPN class did so primarily through *trans*-effects not involving duplications.

This difference between early and later evolutionary mechanisms reflects transition from ‘general’ to more specific solutions: for the DPN class, the general solution of large-scale duplications appears to be the most easily accessible. It arises easily, and is indeed frequently observed during initial selections [30]–[33]. This solution, however, is more difficult to maintain due to pleiotropic effects and the high frequency by which the additional chromosome can be lost, which may explain its absence in species or strains.

For the OPN class, *trans*-effects appear to be the more accessible solution, while chromosome duplications are observed at significantly lower frequency. OPN genes occupy the same chromosomes as DPN genes and thus should have the same likelihood of being duplicated. The preferential modulation of OPN genes by *trans* effects may thus indicate the larger spectrum of *trans* factors that influence the expression of those genes which increases the number of potential *trans* mutations.

Notably, we find that although *trans* mutations arising in our strains did decrease the competitive growth fitness to the same extent as did chromosome duplications, they were easier to maintain. We hypothesize that this reflects the large spectrum of mutations that can compensate for the reduction in cell growth, making it unlikely that the cells precisely revert the mutation leading to the increased expression. This is in contrast to the case of chromosomal duplication, where reversion of the original mutation (loss of the duplicated chromosome) is most likely. Notably, this difference in reversion strategy may explain the prevalence of *trans*-dependent divergence of OPN genes [18]. These *trans* mutations may have arisen during transient selection for high expression, but were maintained even after selection was removed, possibly due to a compensatory mutation. Consistent with this possibility, we recently demonstrated extensive *trans*-dependent expression variability of OPN genes that is buffered by the activity of chromatin regulators [34].

Finally, the most specific solutions (e.g. *cis* regulatory mutations) require more time to emerge compared to the other more general processes, yet their specificity to the gene of interest allows their long-term maintenance in the population which may explain their dominance in the divergence between species [18], [28], [35]. It may also be that those *cis* effects are mostly neutral and do not arise in response to selection, at least not in response to a strong selection. Indeed, if expression can be easily increased through *trans* mutations or duplication, those will arise quickly and will reduce further pressure for the emergence of *cis* mutation.

It should be noted that our study was performed in a haploid background, which favors the emergence of recessive mutations. In contrast, in nature yeast cells exist primarily as diploids. Many of the *trans* mutations we identified will have no effect in a diploid background. Yet, a large fraction of them were only partially recessive and thereby manifested also in the diploid background. It is likely that those mutations will dominate the initial evolution of OPN genes also in a diploid background.

In conclusion, we find that gene expression readily evolves in response to strong selection. Furthermore, we propose that the genetic mechanisms by which expression evolves, and hence the stability of this genetic change, depends on the organization of gene promoter. Together, our study supports the idea that regulatory variance shapes evolutionary path by biasing long-term evolutionary changes to genes with flexible OPN promoter organization.

## Materials and Methods

### Promoter nucleosomes occupancy

To estimate the degree to which each promoter is consistent with the OPN and DPN general classes, we divided the average nucleosome occupancy at the transcription start site (TSS)-proximal region (0–150 bp upstream of TSS) by the average nucleosome occupancy at the TSS-distal region (200–400 bp upstream of the TSS). This measure was averaged over three independent datasets of nucleosome occupancy, including Lee et al. [36], Kaplan et al. [37], and Tsui et al. [38].

### Yeast strains and growth conditions

Selection experiments were performed using the GFP-fusion library strains [24]. BY4741 (MATa his3Δ1 leu2Δ0 met15Δ0 ura3Δ0), was used as a control strain for the CGH analysis. BY4742 (MATα his3Δ1 leu2Δ0 lys2Δ0 ura3Δ0) was used to create diploids in the dominant-recessive experiment as well as to create the mCherry-fusion strains in the *cis*-*trans* experiment. BY4741 MATa YDL227cΔ::TEF2pr-mCherry- kanMX4 was used in the competitive growth experiment. Yeast strains were grown on synthetic complete (SC) media for FACS sorting, flow cytometry analysis and competitive growth experiments. Strains were maintained on YPD-agar plates. Selection for diploids was done on SC-Lys-Met agar plates.

### Selection for high GFP expression

Single colonies of each parental strain were grown to logarithmic phase. Each individual cell in the population was monitored using FACSAriaII cell sorter (Becton Dickinson) using a Coherent Sapphire Solid state 488 nm 20 mW laser. Gating was first done for small, single cell population based on the FSC-A, FSC-W and SSC-A counts, which are associated with cell size and geometry. GFP level for each gated cells was then monitored versus its FSC-A level and cells having the highest GFP versus FSC-A value were collected. In total, 20,000 cells of the top 1.5% (normalized) GFP were collected into 5 ml of SC medium. The selected cells were grown overnight. The population divides for 7–10 times between selections. The population did not reach stationary phase under this conditions so no further dilutions were needed before the next round of selection. Cells were subject to the same selection procedure until a clear shift of the mean expression was observed, or up to a maximum of eleven cycles. The control population went through exactly the same procedure, but 20,000 of the total gated population rather the top 1.5% GFP were collected by FACS. Single colonies were isolated for each evolved strain on YPD-agar plates. Eleven single colonies were picked for each gene and were subject to further analysis.

### Generation of cherry-fused proteins

The mCherry protein was amplified together with the hygromycinB phosphotransferase (hph), a gene conferring resistance to the antibiotic hygromycin B, from the pBS35 plasmid using the primers F2 and R1 used in the construction of the GFP library (http://yeastgfp.yeastgenome.org/yeastGFPOligoSequence.txt). The amplified fragments were transformed into the yeast strain BY4742 using the LiAc/SS-Carrier/PEG transformation method [39]. After overnight recovery, the yeast cells were plated on synthetic complete (SC) medium+hygromycin B (0.3 mg/ml, Calbiochem). Correct integration was verified by PCR using cherry reverse primer (5′-tgaactccttgatgatggcc-3′) and gene specific check primer (GFP library). The expression level of each fusion protein was measured prior to mating with its GFP homologue.

### Measurements of fluorescence using flow cytometry, analysis of data

Fluorescence was measured by flow cytometery on the BD LSRII system (BD Biosciences) with a High Throughput Sampler extension (HTS), With Excitation wavelength of 488 nm for GFP and of 594 nm for Cherry. The FACS fcs files were imported into Matlab using an available script [40]. The FACS data was processed by gating based on FSC and SSC, removal of outliers from the GFP population and calculation of mean and standard deviation.

### Quantitative PCR

DNA from 3 individual colonies of each evolved strain analyzed as well as from its parental strain at two replicates. DNA was extracted using Masterpure Yeast DNA Purification Kit (Epicentre Biotechnologies). Real time PCR was performed in Lightcycler 480 (Roche). Reactions were done using LightCycler 480 Probes Master. GFP DNA content was detected using primers: 5′-cacatggtccttcttgagtttg-3′ and 5′-atagttcatccatgccatgtgta-3′ together with probe no. 3 (Universal ProbeLibrary, Roche) Act1 was used as a reference gene and was detected using primers: 5′-tccgtctggattggtggtand-3′ and 5′-tgagatccacatttgttggaag-3′ together with probe no 139 (Universal ProbeLibrary, Roche). GFP gene content of each strain was normalized to its parental strain. Analysis of the results was done using LightCycler 480 software. DNA content of the reverted strains was monitored in two individual colonies together with the corresponding evolved strain. Real time reaction were done using Absolute Blue SYBR Green mix (Thermo Scientific), analyzed and normalized as above. Primer used were: YBR197C (chr 2) 5′-aggtgaaagtaagcgacgcg-3′; 5′-tgaaccagctgagggtttcct-3′ YCR047C (chr 3) 5′-tatgtcgtccacctggtcgtcg-3′; 5′tcctaaacagcggttgatgagg3′ ERG1 (chr 7) 5′- cagtcataccaccaccagtcaatg-3′; 5′-gccaaactcctacttgccagc-3′ URA1 (chr 11) 5′- tccaagatagcgaattcaacg-3′; 5′-tttcccaggcacattaggac-3′ SMA2 (chr 13) 5′- acctaccgtttggcattgac-3′; 5′-atagggcatttcctgtgtgc-3′ GFP 5′- gtggagagggtgaaggtga-3′; 5′- gttggccatggaacaggtag-3′ ACT1 5′- tcgttccaatttacgctggtt-3′; 5′ –cgattctcaaaatggcgtg-3′.

### Comparative genomic hybridization

DNA was extracted using Masterpure Yeast DNA Purification Kit (Epicentre Biotechnologies). After RNAse treatment and EtOH precipitation, DNA was digested using AluI and RsaI restriction enzymes (Promega) and purified with QIAquick PCR purification kit (QIAGEN). DNA was then labeled and Hybridized to microarrays following Agilent Oligonucleotide Array-Based CGH for Genomic DNA Analysis protocol. Arrays were scanned using Agilent microarray scanner and quantified using the Spotreader software (Niles Scientific).

### Microarray design

A 180K custom Agilent CGH microarray was defined by selecting 60,000 high-quality probes (with average spacing of 140 bp) from the Agilent-014741 Yeast Whole Genome 244K microarray design. Three repeats of each selected probes were dispersed at different random positions in the microarray.

### CGH data analysis

Probes with CV>40% or median<3000 were removed. The signal was calculated as log of (median intensity – median background intensity). Negative values were removed. The repeats were averaged. We have noted that the averaged signal was negatively correlated with distance from telomeres (r = −0.24), and therefore subtracted the lowess curve of signal as a function of distance (matlab malowess function span of 10%). The signal was further normalized by subtracting lowess curve (span = 1%) of the reference (Cy3 against Cy3 signal of WT, and Cy5 against Cy5 signal of WT). The signal shown in both unsmoothed and smoothed (lowess span = 0.5%) forms.

### Competitive growth

To characterize the competitive growth of each of the strains, we utilize a high throughput flow cytometry assay. For each strain tested two evolved colonies as well as two colonies of the corresponding parental strain were analyzed. Each colony was grown together with a wild type strain (BY4741) marked with mCherry expressed under the constitutive TEF promoter in the same well of a 96-well plate. The strains were inoculated in the well in equal concentrations and diluted repeatedly in 24 hour intervals for 4 days. GFP and mCherry cells frequencies were measured by FACS at the initial inoculation and at each dilution point. Each experiment was repeated 3 times. The differences in the strains growth rate can be derived from the frequencies measured by FACS. The fitness advantage of one competing strain over the other is calculated as follows: Denote ni as the number of cells of type i, gi as the growth rate of cell type i, fi as the frequency of cells type i out of the whole population, and p as the number of types of cells. If the frequencies of any two types of cells are divided, log2 transformed, and plotted against time, we get a curve whose slope is the difference in their growth rate, and this is what we refer to as fitness advantage:
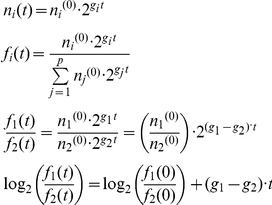



## Supporting Information

Figure S1 Nucleosome occupancy measure: Black lines denote the average nucleosome occupancy, with occupancy score given in brackets (Materials and Methods). The average occupancy for all genes classified as DPN or OPN is shown in green and red, respectively. All genes in our study are shown.
(PDF)

Figure S2 Increased expression in response to selection: Shown is the median GFP level relative to the parental strain at successive days of selection. Note the different Y scale for TKL1.
(PDF)

Figure S3 Classifying mutations into dominant versus recessive: Eleven single colonies were isolated from each evolved strain and the underlying mutation was classified as dominant versus recessive by mating to WT strain. Red circles are the relative increase in fluorescence in the haploids whereas blue dots denote the fluorescence of the respective heterozygote diploids. Values are normalized to the fluorescence of the parental strain (or the parental mated with a wild-type strain for the diploids). Note that the initial classification of evolved strains was done based on the analysis of the single colonies presented here, as follow: To select promoters that underwent evolution we have tested whether the fluorescence of colonies after the evolution differs significantly from its initial value. To this end we determined how many of the 11 single colonies isolated for each strain differ significantly from the fluorescence of the unevolved strain. The significance of the change was estimated as follows: log2(normalized fluorescence) of 55 single colonies of control populations taken from five different promoters were measured and estimated to be distributed approximately N(−0.031, 0.138). A change was considered significant if its' p-value was less than 0.001.
(PDF)

Figure S4 Classifying mutations into *cis* versus *trans*: For each evolved strain, we generated a wild-type strain in which the corresponding protein is fused to the mCherry marker. This strain was then mated with the ancestor strain (carrying the non-evolved GFP marker) and to the evolved strain (in which GFP fluorescence was higher). We asked whether mating with the evolved strain will increase also the mCherry fluorescence (*trans* mutation) or not (*cis* mutation). Shown is the increase in GFP fluorescence versus the increase in mCherry fluorescence in the heterozygote (wt×evolved) strains, relative to the parental (wt×ancestral) strain. Note that in some cases (e.g. NSR1) two types of colonies are presented. Some dominant strains were omitted from this analysis due to low levels of mCherry expression. Coordinated changes in the two alleles (*trans* mutation) is seen in the cases in which expression in a diploid background is reduced compared to the haploid background but remains higher than the expression of the non-evolved strain. In those cases, the mCherry marker increases in expression upon mating to the evolved strain to an extent similar to the GFP marker. Note also that for some of the recessive mutations, the increased expression of the evolved strain is lost upon mating with a wild-type strain, and hence in our experiment both markers show only the wild-type expression levels.
(PDF)

Figure S5 Identifying large-scale duplications: CGH analysis was performed to define genomic rearrangements in the evolved strains. Shown are the hybridization ratios (relative to a wt strain) of probes ordered by their genomic location. Vertical bars mark chromosome ends. The genomic location of the selected gene is shown in brackets. Note that for GUS1, the increase in signal ratio is less than two fold, perhaps indicating a rapid loss of the duplicated chromosome during the course of the experiment resulting in a mixed population.
(PDF)

Figure S6 Aneuploid strains revert more rapidly in the absence of selection: Nineteen of the evolved strains were diluted repeatedly in SC for ∼130 generations. The temporal change in fluorescence levels (normalized to its value in the parental strain) is shown.
(PDF)

Figure S7 Evolved strains show some growth defects. The relative fitness of each strain before and after evolution are plotted. For details see Material and Methods.
(PDF)

Table S1 Genes used in our study and their classifications.
(PDF)
