# Supplementary figures and images for: Promoter Nucleosome Organization Shapes the Evolution of Gene Expression
Source: PLoS Genet. 2012 Mar 15;8(3):e1002579. doi: 10.1371/journal.pgen.1002579 (PMC3305400; doi:10.1371/journal.pgen.1002579)

Fig. S1

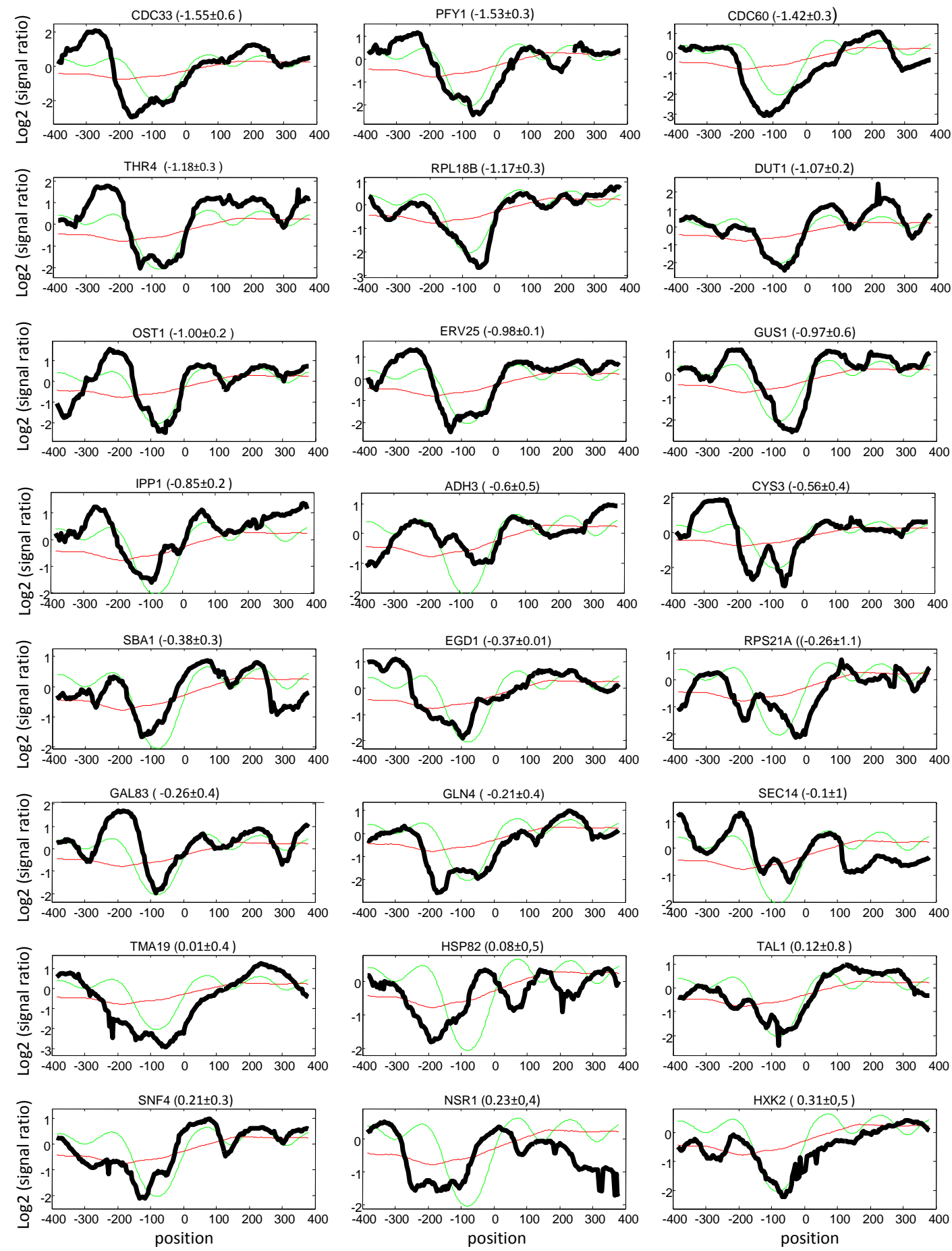

Fig. S1

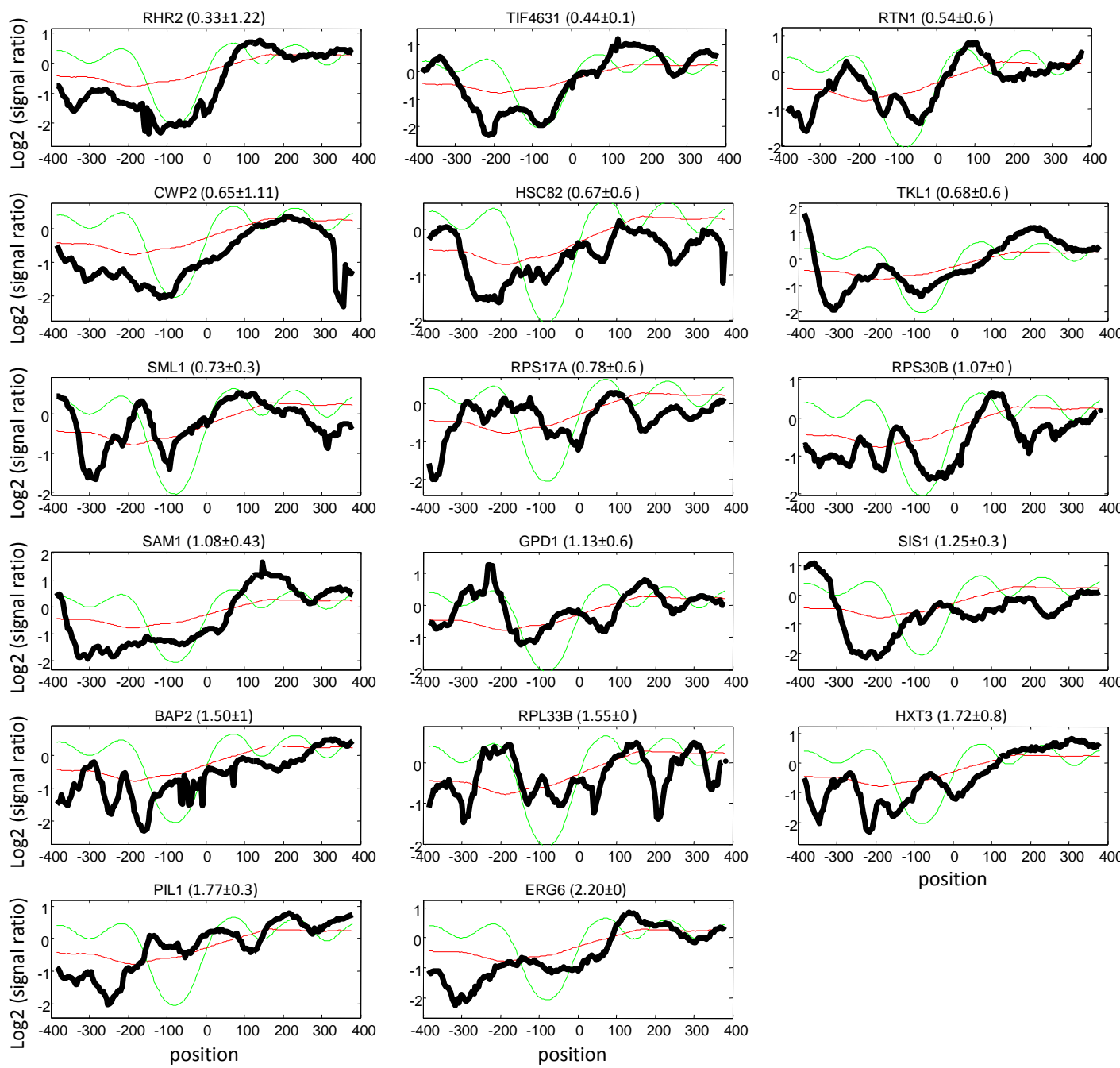

Supplement: Figure S1 — Nucleosome occupancy measure: Black lines denote the average nucleosome occupancy, with occupancy score given in brackets (Materials and Methods). The average occupancy for all genes classified as DPN or OPN is shown in green and red, respectively. All genes in our study are shown. (PDF) [file pgen.1002579.s001.pdf]

Fig. S2

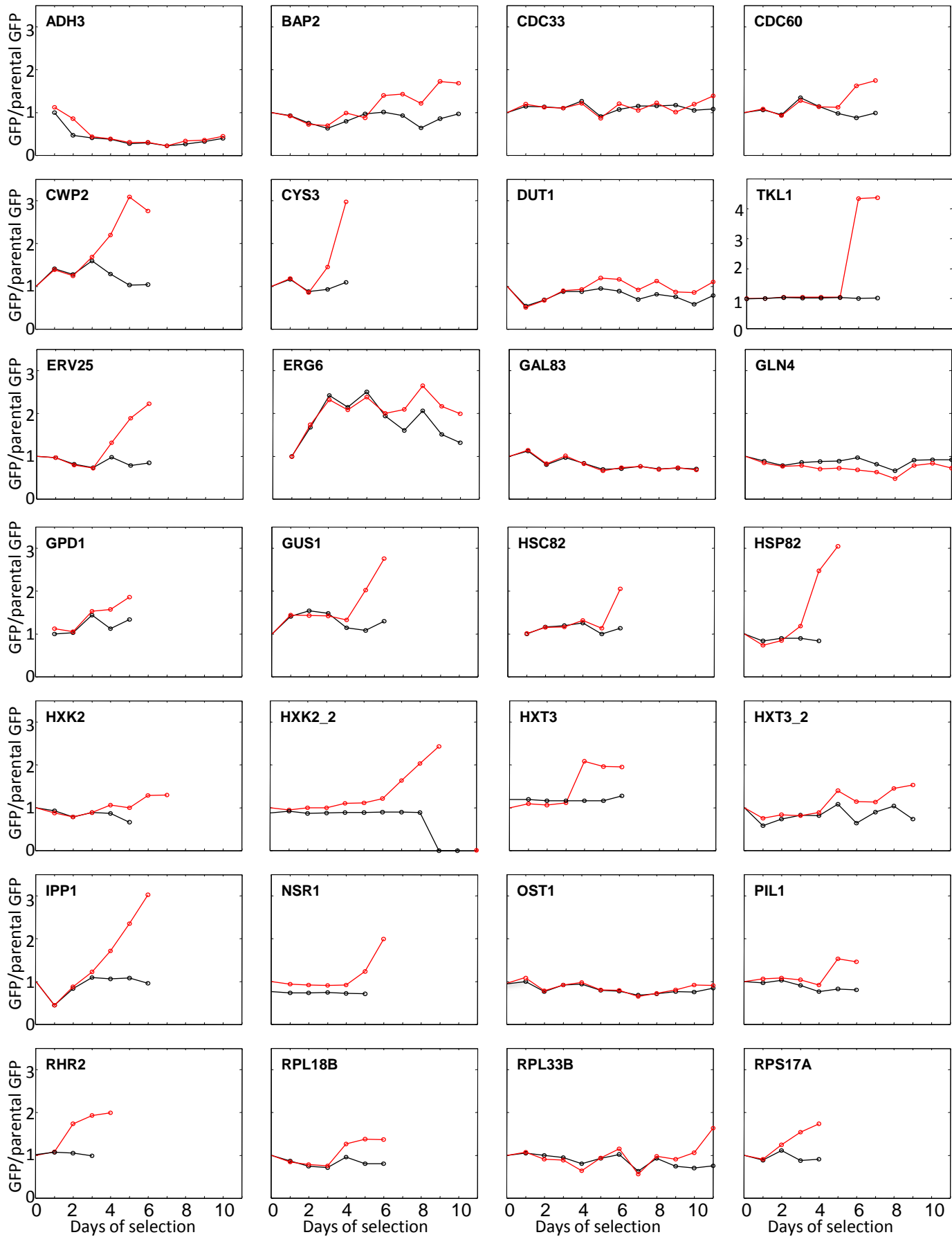

Fig S2

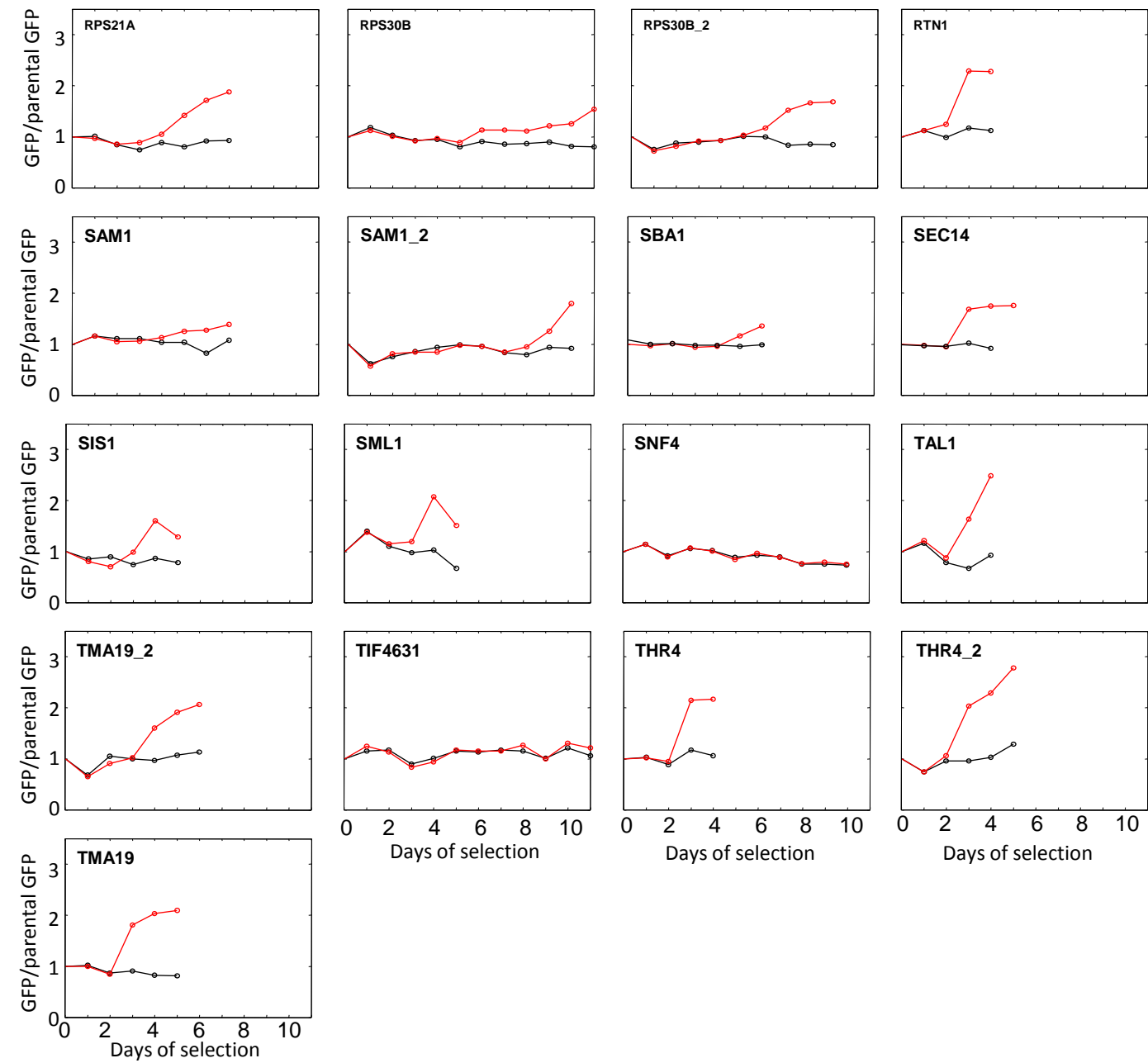

Supplement: Figure S2 — Increased expression in response to selection: Shown is the median GFP level relative to the parental strain at successive days of selection. Note the different Y scale for TKL1. (PDF) [file pgen.1002579.s002.pdf]

Fig S4

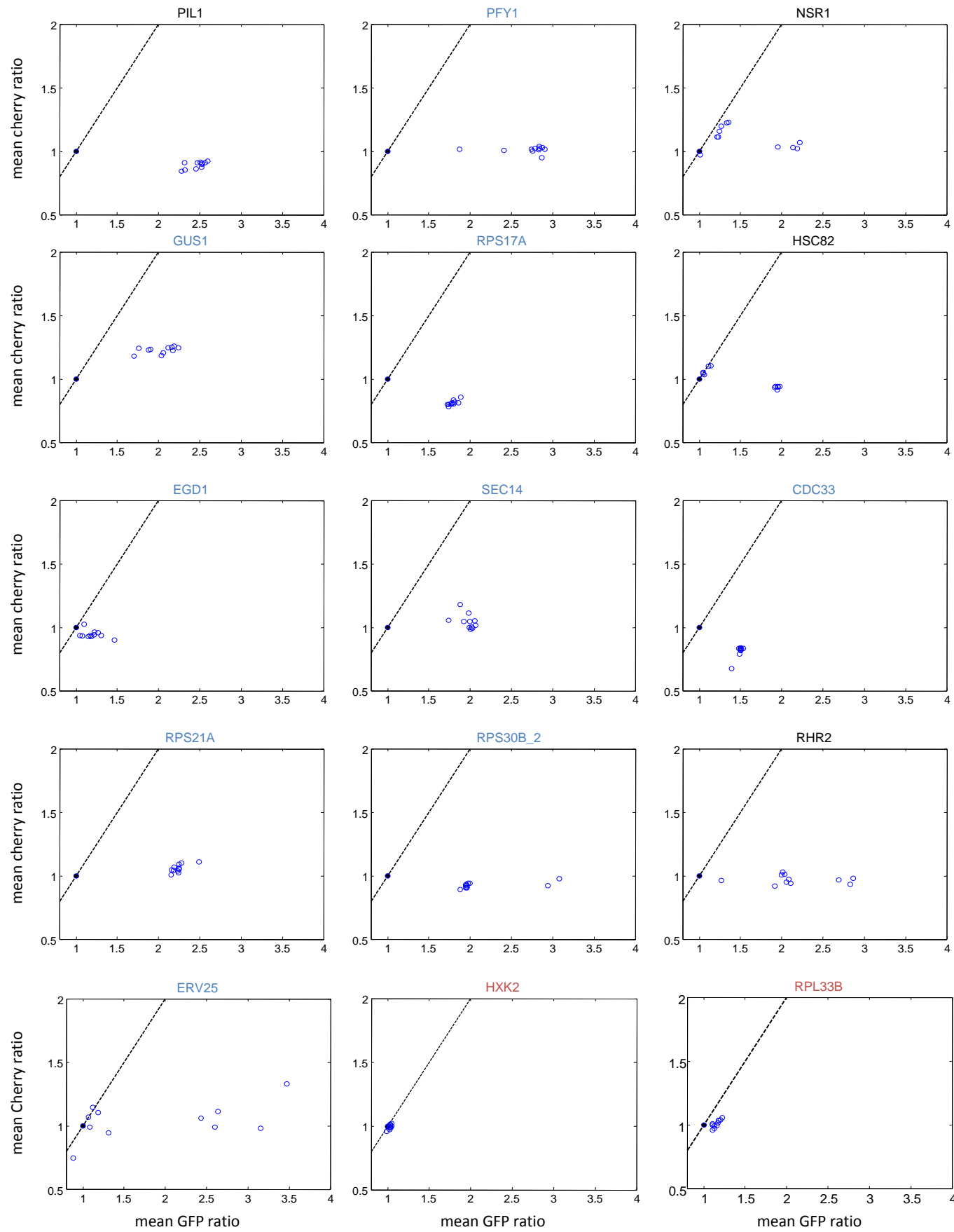

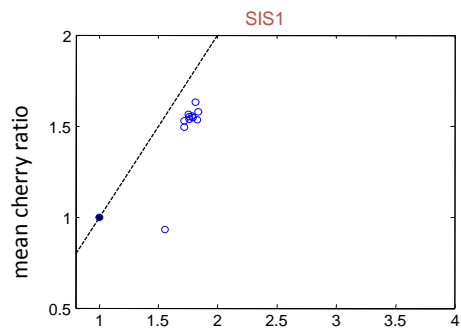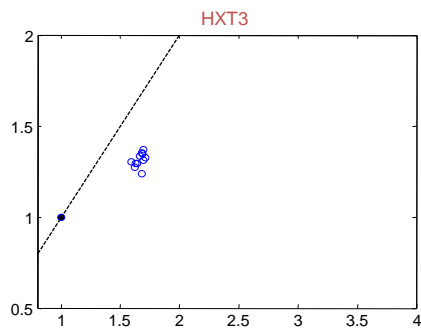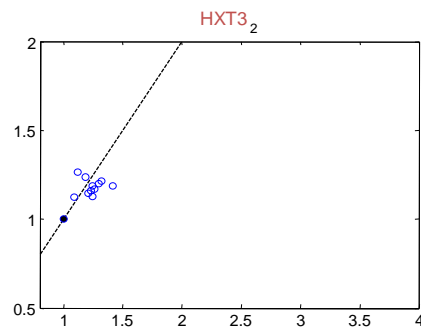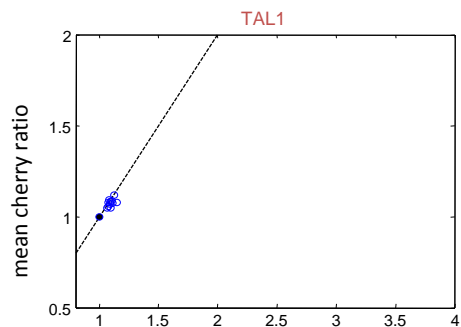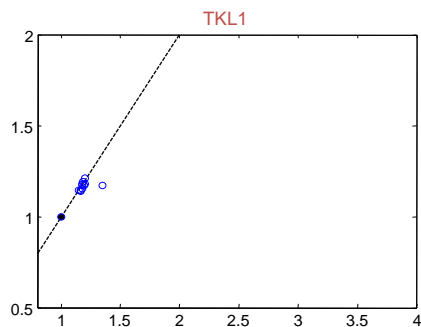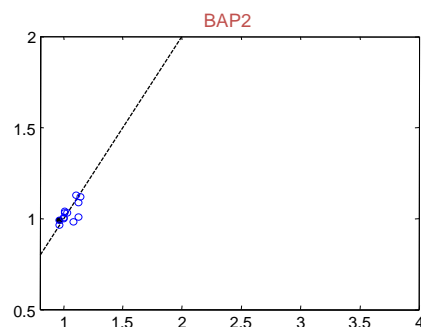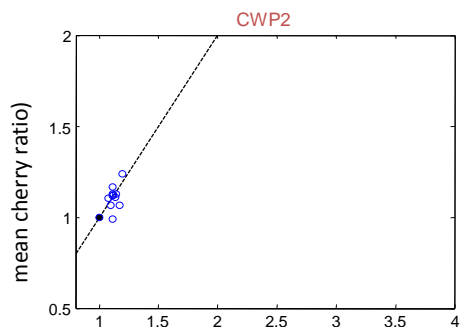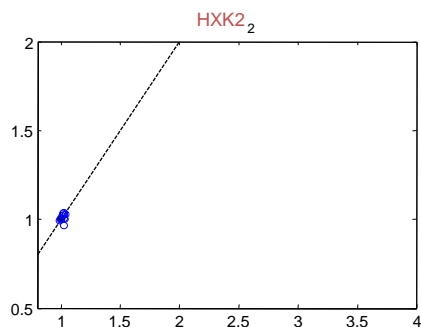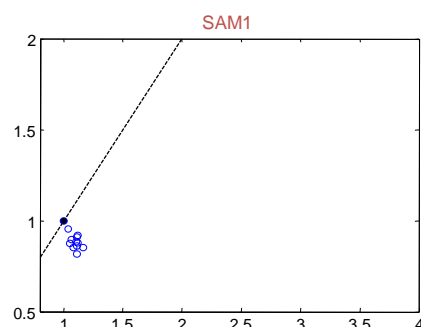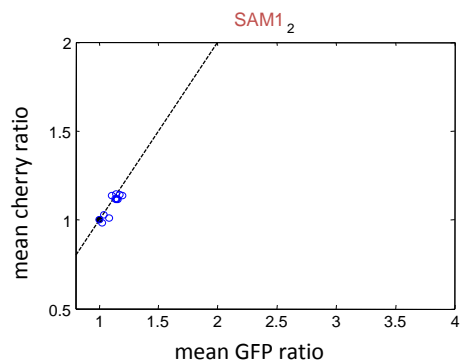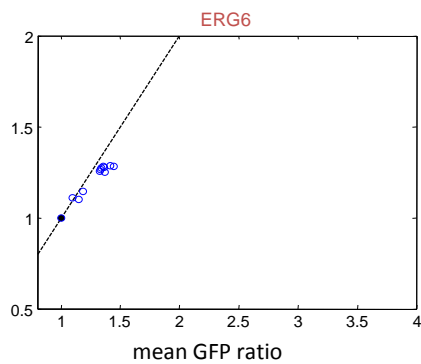

Supplement: Figure S4 — Classifying mutations into cis versus trans: For each evolved strain, we generated a wild-type strain in which the corresponding protein is fused to the mCherry marker. This strain was then mated with the ancestor strain (carrying the non-evolved GFP marker) and to the evolved strain (in which GFP fluorescence was higher). We asked whether mating with the evolved strain will increase also the mCherry fluorescence (trans mutation) or not (cis mutation). Shown is the increase in GFP fluorescence versus the increase in mCherry fluorescence in the heterozygote (wt×evolved) strains, relative to the parental (wt×ancestral) strain. Note that in some cases (e.g. NSR1) two types of colonies are presented. Some dominant strains were omitted from this analysis due to low levels of mCherry expression. Coordinated changes in the two alleles (trans mutation) is seen in the cases in which expression in a diploid background is reduced compared to the haploid background but remains higher than the expression of the non-evolved strain. In those cases, the mCherry marker increases in expression upon mating to the evolved strain to an extent similar to the GFP marker. Note also that for some of the recessive mutations, the increased expression of the evolved strain is lost upon mating with a wild-type strain, and hence in our experiment both markers show only the wild-type expression levels. (PDF) [file pgen.1002579.s004.pdf]

Fig. S5

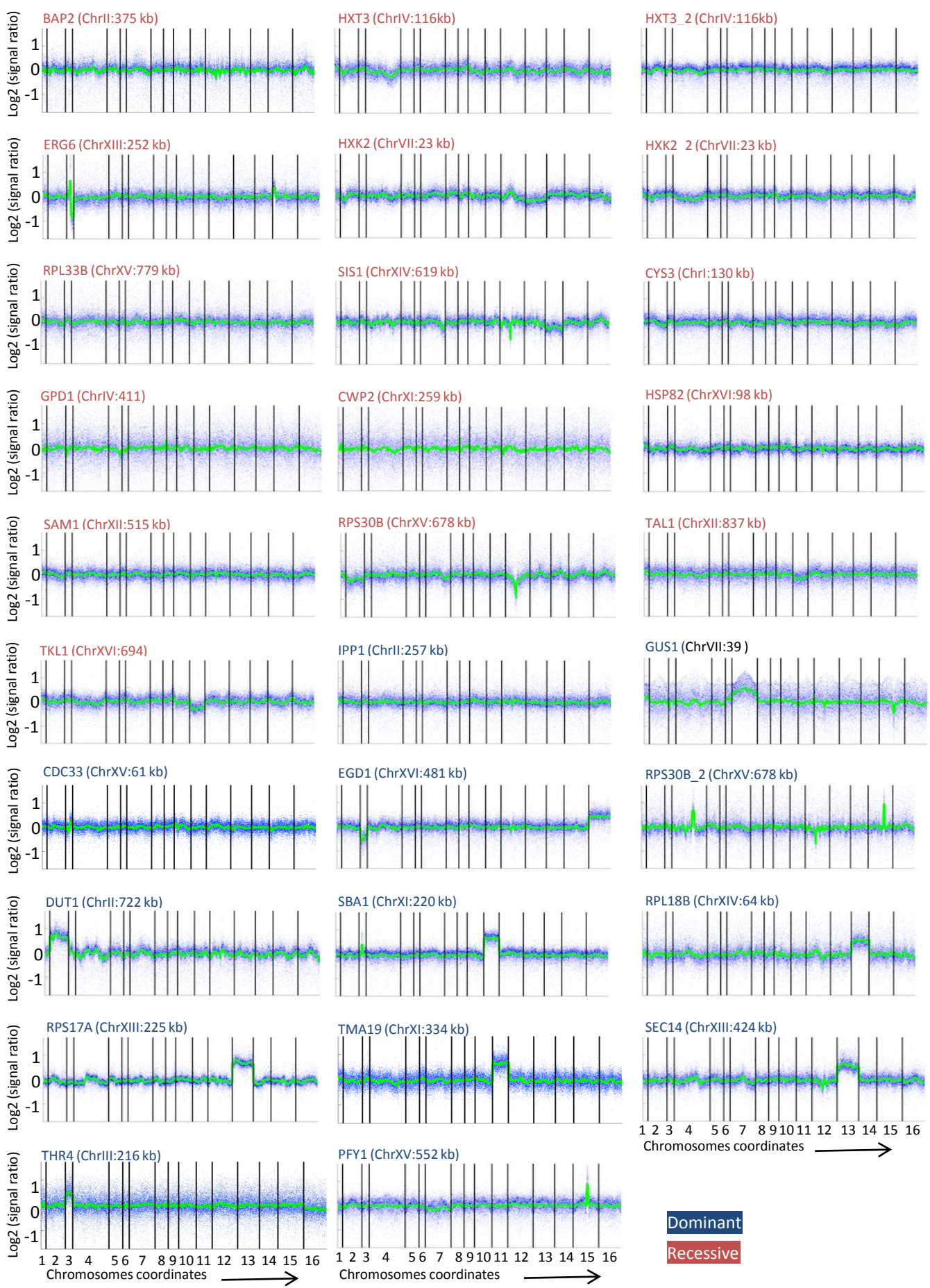

Supplement: Figure S5 — Identifying large-scale duplications: CGH analysis was performed to define genomic rearrangements in the evolved strains. Shown are the hybridization ratios (relative to a wt strain) of probes ordered by their genomic location. Vertical bars mark chromosome ends. The genomic location of the selected gene is shown in brackets. Note that for GUS1, the increase in signal ratio is less than two fold, perhaps indicating a rapid loss of the duplicated chromosome during the course of the experiment resulting in a mixed population. (PDF) [file pgen.1002579.s005.pdf]

Fig. S6

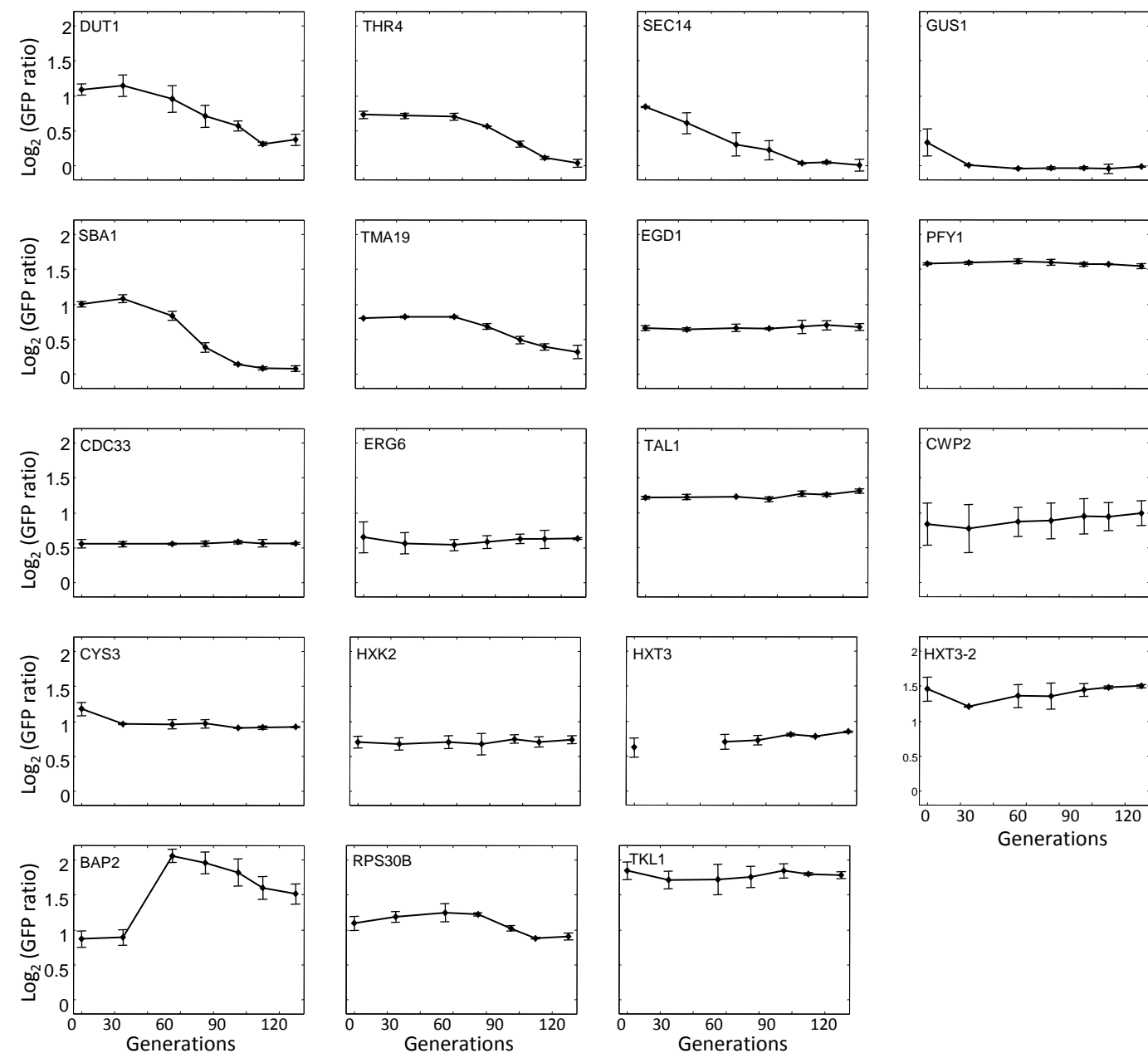

Supplement: Figure S6 — Aneuploid strains revert more rapidly in the absence of selection: Nineteen of the evolved strains were diluted repeatedly in SC for ∼130 generations. The temporal change in fluorescence levels (normalized to its value in the parental strain) is shown. (PDF) [file pgen.1002579.s006.pdf]

Fig. S7

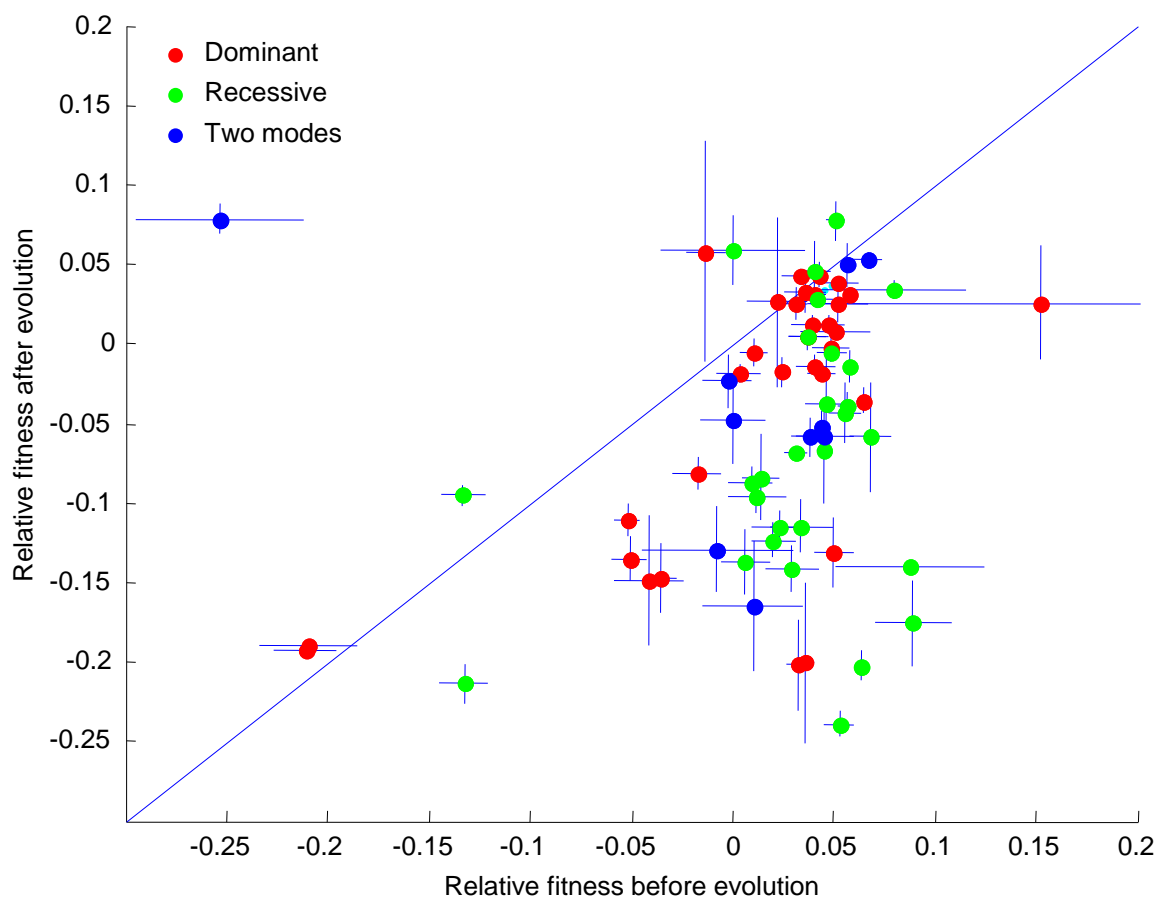

Supplement: Figure S7 — Evolved strains show some growth defects. The relative fitness of each strain before and after evolution are plotted. For details see Material and Methods. (PDF) [file pgen.1002579.s007.pdf]
