# Supplementary material for: Promoter Nucleosome Organization Shapes the Evolution of Gene Expression
Source: PLoS Genet. 2012 Mar 15;8(3):e1002579. doi: 10.1371/journal.pgen.1002579 (PMC3305400; doi:10.1371/journal.pgen.1002579)
Supplement: Figure S3 — Classifying mutations into dominant versus recessive: Eleven single colonies were isolated from each evolved strain and the underlying mutation was classified as dominant versus recessive by mating to WT strain. Red circles are the relative increase in fluorescence in the haploids whereas blue dots denote the fluorescence of the respective heterozygote diploids. Values are normalized to the fluorescence of the parental strain (or the parental mated with a wild-type strain for the diploids). Note that the initial classification of evolved strains was done based on the analysis of the single colonies presented here, as follow: To select promoters that underwent evolution we have tested whether the fluorescence of colonies after the evolution differs significantly from its initial value. To this end we determined how many of the 11 single colonies isolated for each strain differ significantly from the fluorescence of the unevolved strain. The significance of the change was estimated as follows: log2(normalized fluorescence) of 55 single colonies of control populations taken from five different promoters were measured and estimated to be distributed approximately N(−0.031, 0.138). A change was considered significant if its' p-value was less than 0.001. (PDF) [file pgen.1002579.s003.pdf]

Fig. S3

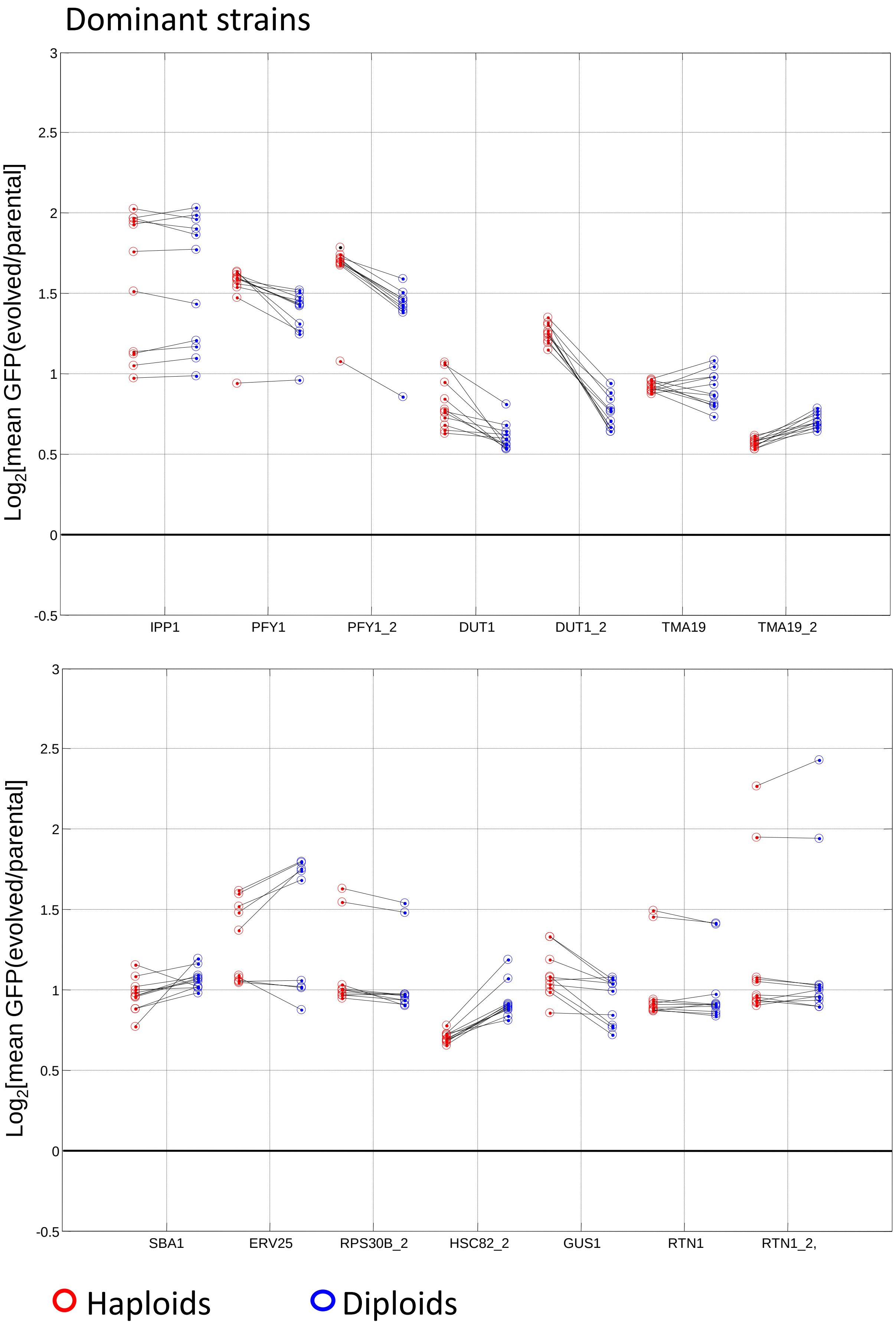

Dominant strains
Log2[mean GFP(evolved/parental)]
3
2.5
2
1.5
1
0.5
0
-0.5
IPP1
PFY1
PFY1_2
DUT1
DUT1_2
TMA19
TMA19_2
Log2[mean GFP(evolved/parental)]
3
2.5
2
1.5
1
0.5
0
-0.5
SBA1
ERV25
RPS30B_2
HSC82_2
GUS1
RTN1
RTN1_2,
Haploids
Diploids

Fig. S3

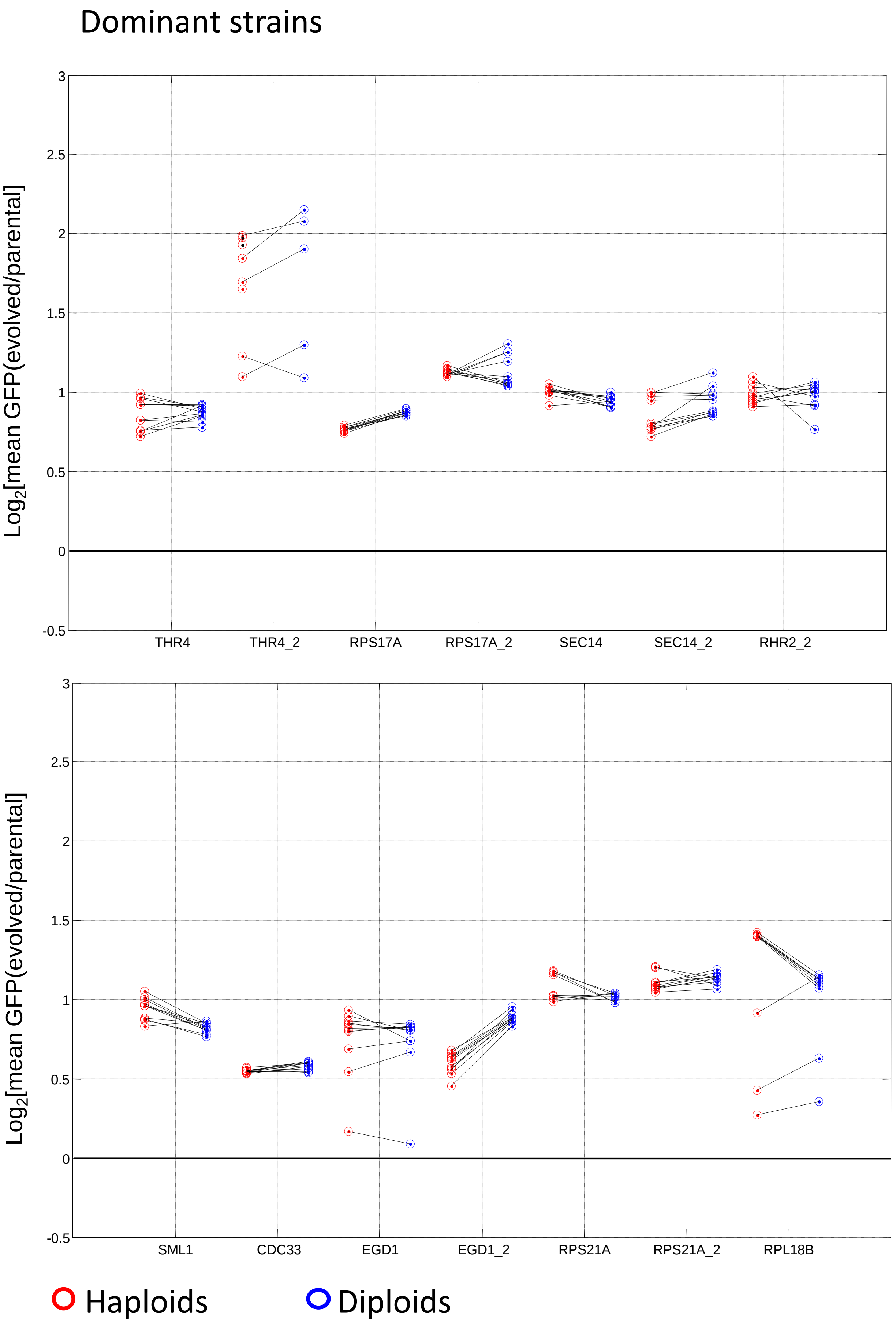
Dominant strains
Log2[mean GFP(evolved/parental]
3
2.5
2
1.5
1
0.5
0
-0.5
THR4
THR4_2
RPS17A
RPS17A_2
SEC14
SEC14_2
RHR2_2
Log2[mean GFP(evolved/parental]
3
2.5
2
1.5
1
0.5
0
-0.5
SML1
CDC33
EGD1
EGD1_2
RPS21A
RPS21A_2
RPL18B
Haploids
Diploids

Fig. S3

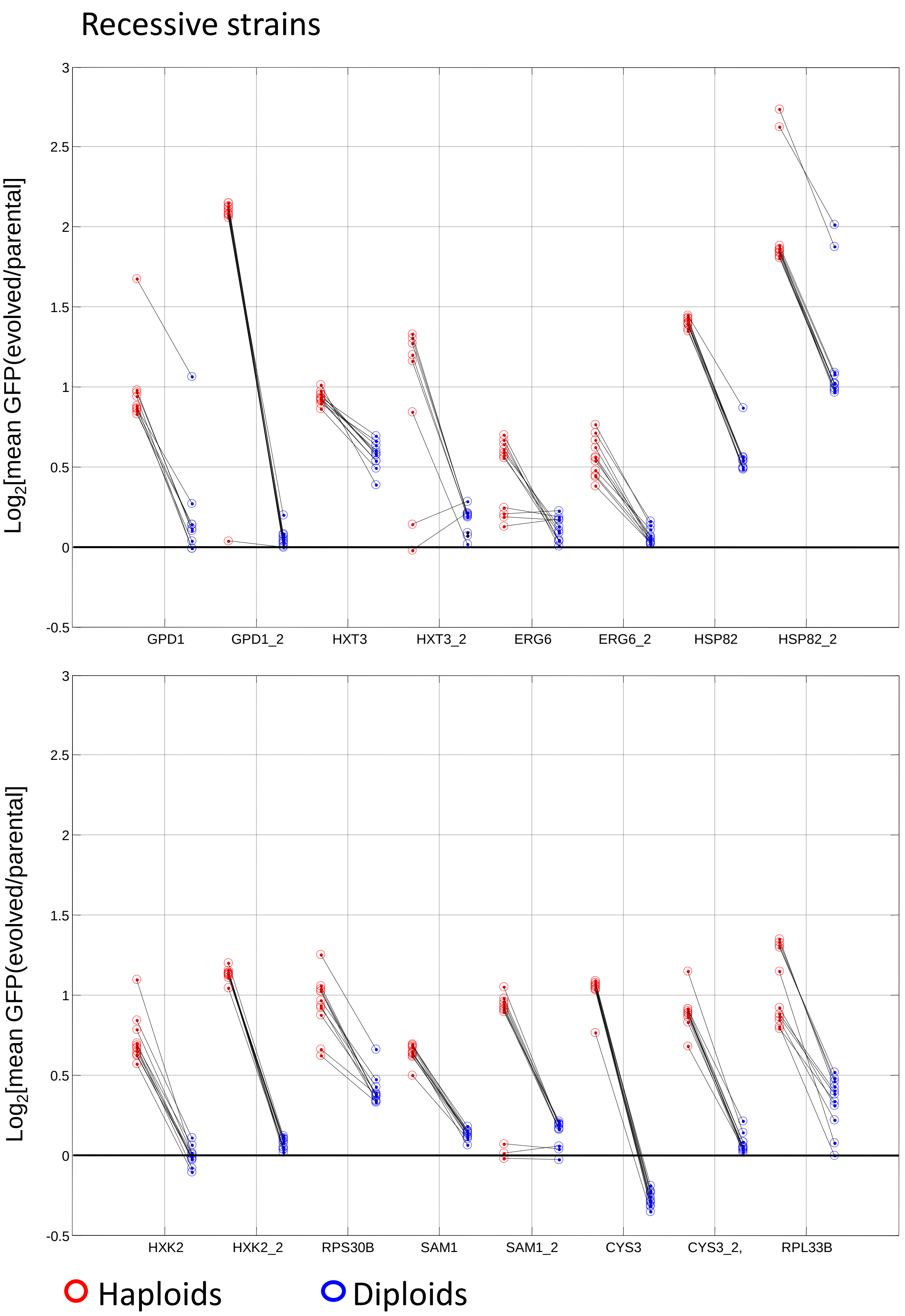

Recessive strains
Log2[mean GFP(evolved/parental]
GPD1
GPD1_2
HXT3
HXT3_2
ERG6
ERG6_2
HSP82
HSP82_2
HXK2
HXK2_2
RPS30B
SAM1
SAM1_2
CYS3
CYS3_2,
RPL33B
3
2.5
2
1.5
1
0.5
0
-0.5
Haploids
Diploids

Fig. S3

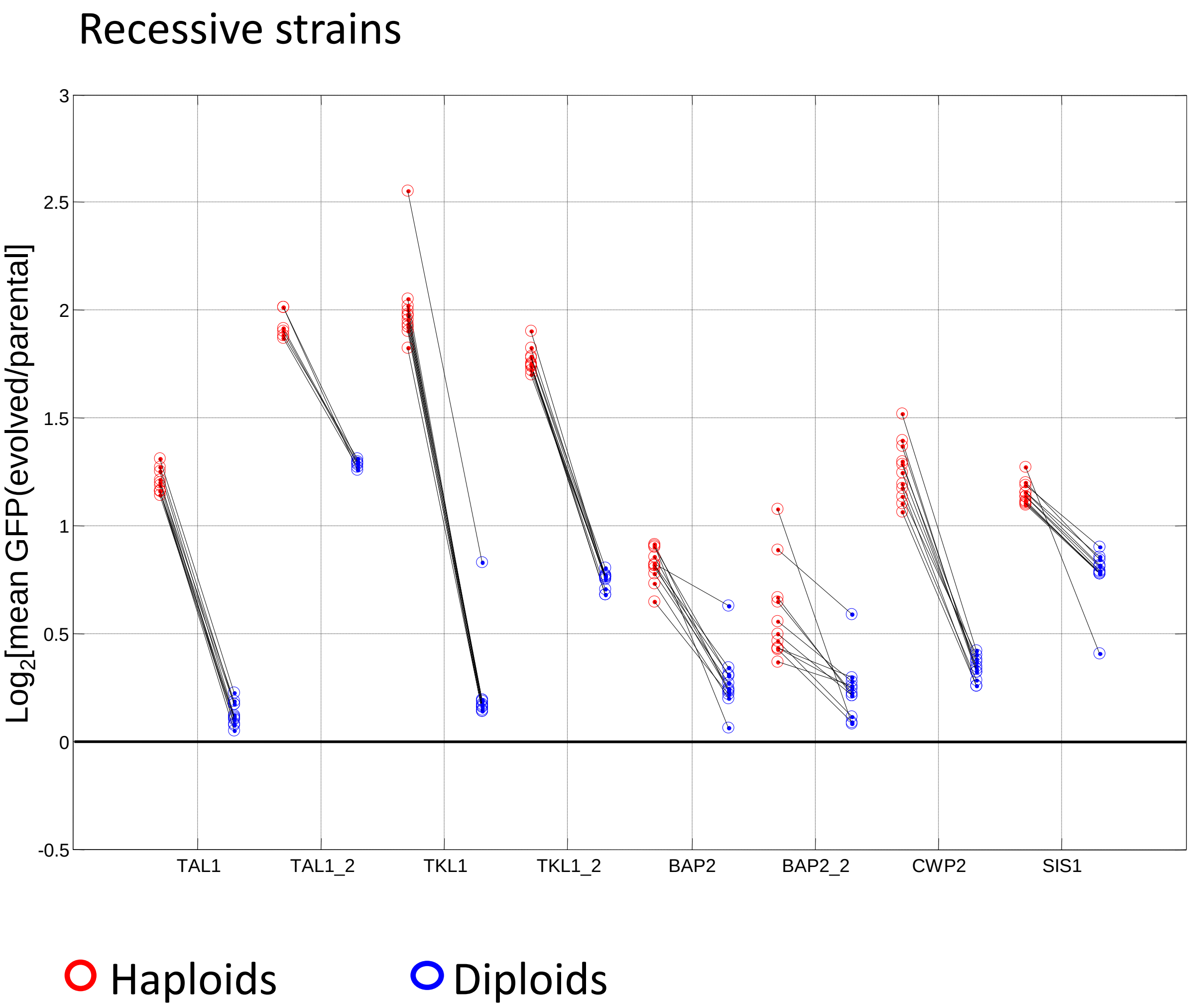
Recessive strains
Log2[mean GFP(evolved/parental)]
3
2.5
2
1.5
1
0.5
0
-0.5
TAL1
TAL1_2
TKL1
TKL1_2
BAP2
BAP2_2
CWP2
SIS1
Haploids
Diploids

Fig. S3

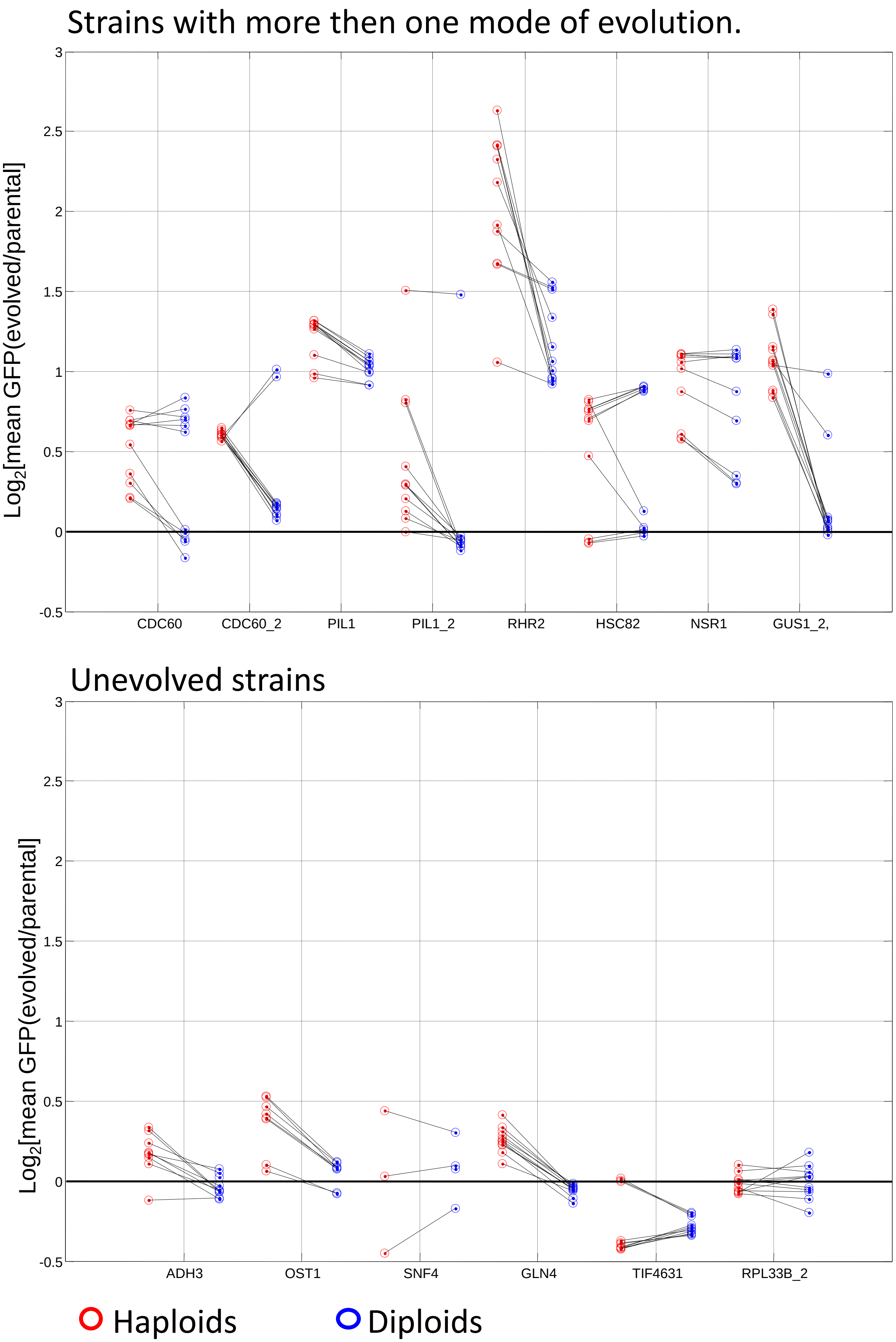

Strains with more then one mode of evolution.
Log2[mean GFP(evolved/parental]
3
2.5
2
1.5
1
0.5
0
-0.5
CDC60
CDC60_2
PIL1
PIL1_2
RHR2
HSC82
NSR1
GUS1_2,
Unevolved strains
Log2[mean GFP(evolved/parental]
3
2.5
2
1.5
1
0.5
0
-0.5
ADH3
OST1
SNF4
GLN4
TIF4631
RPL33B_2
Haploids
Diploids
