# Supplementary material for: Promoter Nucleosome Organization Shapes the Evolution of Gene Expression
Source: PLoS Genet. 2012 Mar 15;8(3):e1002579. doi: 10.1371/journal.pgen.1002579 (PMC3305400; doi:10.1371/journal.pgen.1002579)
Supplement: Table S1 — Genes used in our study and their classifications. (PDF) [file pgen.1002579.s008.pdf]

Name	systematic name	Av.Nuc.Occ ratio[a]	Initial expression	Initial noise (η)[b]	Distance from telomere	TATA[c]	Repeats[c]	Mode of Evolution (1st	Mode of Evolution (2 nd round)	Duplication	reversion	comments	Gene description
CDC33	YOL139C	-1.55±0.6	1700	0.12	60.38 Kb	-1		Dominant		no	no	diploids (also original strain)	Cytoplasmic mRNA cap binding protein and translation initiation factor eIF4E
PFY1	YOR122C	-1.53±0.3	500	0.16	538.40 Kb	0		Dominant	Dominant	yes	no		Profilin, binds actin, phosphatidylinositol 4,5-bisphosphate, and polyproline regions
CDC60	YPL160W	-1.42±0.3	1200	0.16	246.99 Kb	0		Two modes	Two modes	Yes (dominant colonies)			Cytosolic leucyl tRNA synthetase
THR4	YCR053W	-1.18±0.3	1400	0.13	98.38 Kb	0		Dominant	Dominant	yes	yes		Threonine synthase
RPL18B	YNL301C	-1.17±0.3	1000	0.19	63.57 Kb	-1		Dominant		yes			Protein component of the large (60S) ribosomal subunit
DUT1	YBR252W	-1.07±0.2	100	0.39	90.13 Kb	0		Dominant	Dominant	yes	yes		deoxyuridine triphosphate diphosphatase (dUTPase)
OST1	YJL002C	-1.00±0.2	300	0.16	311.10 Kb	-1		Did not evolve					Alpha subunit of the oligosaccharyltransferase complex of the ER lumen
ERV25	YML012W	-0.98±0.1	500	0.18	246.12 Kb	-1		Dominant		yes		mix of diploids and haploids after evolut	member of the p24 family involved in endoplasmic reticulum to Golgi transport
GUS1	YGL245W	-0.97±0.6	800	0.15	39.02 Kb	0		Dominant	Two modes	yes	yes		Glutamyl-tRNA synthetase
IPP1	YBR011C	-0.85±0.2	500	0.23	257.11 Kb	-1		Dominant		no			Cytoplasmic inorganic pyrophosphatase (PPase)
ADH3	YER027C	-0.6±0.5	200	0.45	208.98 Kb	-1		Did not evolve					Mitochondrial alcohol dehydrogenase isozyme III
CYS3	YAL012W	-0.56±0.4	1000	0.15	98.22 Kb	-1		Recessive	Recessive	no	no		Cystathionine gamma-lyase
SBA1	YKL117W	-0.38±0.3	700	0.17	219.97 Kb	-1		Dominant		yes	yes		Co-chaperone that binds to and regulates Hsp90 family chaperones
EGD1	YPL037C	-0.37±0.01	1500	0.15	466.16 Kb	0		Dominant	Dominant	yes	no		Subunit beta1 of the nascent polypeptide-associated complex (NAC) involved in protein targeting
RPS21A	YKR057W	-0.26±1.1	1400	0.12	114.57 Kb	-1		Dominant	Dominant	yes			Protein component of the small (40S) ribosomal subunit
GAL83	YGL115W	-0.26±0.4	100	0.37	292.04 Kb	1		Did not evolve					One of three possible beta-subunits of the Snf1 kinase complex
GLN4	YOR168W	-0.21±0.4	900	0.18	439.56 Kb	1		Did not evolve					Glutamine tRNA synthetase
SEC14	YMR079W	-0.1±1	600	0.14	424.99 Kb	-1		Dominant	Dominant	yes	yes		Phosphatidylinositol/phosphatidylcholine transfer protein
TMA19	YKL056C	0.01±0.4	2000	0.15	331.89 Kb	1	1	Dominant	Dominant	yes	yes		Protein that associates with ribosomes
HSP82	YPL240C	0.08±0,5	2600	0.28	96.50 Kb	-1		Recessive	Recessive	no			Hsp90 chaperone
TAL1	YLR354C	0.12±0.8	600	0.19	240.82 Kb	0	1	Recessive	Recessive	no	no		Transaldolase, enzyme in the non-oxidative pentose phosphate pathway
SNF4	YMR083W	0.21±0.3	100	0.23	434.79 Kb	-1		Did not evolve					Activating gamma subunit of the AMP-activated Snf1p kinase complex
NSR1	YGR159C	0.23±0,4	900	0.20	283.29 Kb	-1		Two modes		Yes (dominant colonies)			Nucleolar protein that binds nuclear localization sequences
HXK2	YGL253W	0.31±0,5	3100	0.18	23.93 Kb	-1	1	Recessive	Recessive	no	no		Hexokinase isoenzyme 2 that catalyzes phosphorylation of glucose in the cytosol
RHR2	YIL053W	0.33±1.22	2400	0.24	184.02 Kb	0	1	Two modes	Dominant	Yes (dominant colonies)			Constitutively expressed isoform of DL-glycerol-3-phosphatase
TIF4631	YGR162W	0.44±0.1	1200	0.17	264.02 Kb	-1		Did not evolve					Translation initiation factor eIF4G
RTN1	YDR233C	0.54±0.6	1000	0.14	601.57 Kb	0		Dominant	Dominant	yes	.		ER membrane protein that interacts with Sey1p to maintain ER morphology
CWP2	YKL096W-A	0.65±1.11	900	0.22	258.90 Kb	0		Recessive		no	no		Covalently linked cell wall mannoprotein, major constituent of the cell wall
HSC82	YMR186W	0.67±0.6	5600	0.22	289.96 Kb	1		Two modes	Dominant	Yes (dominant colonies)			Cytoplasmic chaperone of the Hsp90 family
TKL1	YPR074C	0.68±0.6	2500	0.17	253.23 Kb	0		Recessive	Recessive	no	no		Transketolase,enzyme in the non-oxidative pentose phosphate pathway
SML1	YML058W	0.73±0.3	800	0.24	159.38 Kb	1		Dominant		yes			Ribonucleotide reductase inhibitor involved in regulating dNTP production
RPS17A	YML024W	0.78±0.6	1700	0.14	225.89 Kb	-1	1	Dominant	Dominant	yes			Ribosomal protein 51 (rp51) of the small (40s) subunit
RPS30B	YOR182C	1.07±0	1300	0.15	412.50 Kb			Recessive	Dominant	no	no		Protein component of the small (40S) ribosomal subunit
SAM1	YLR180W	1.08±0.43	2000	0.16	515.26 Kb	-1		Recessive	Recessive	no			S-adenosylmethionine synthetase
GPD1	YDL022W	1.13±0.6	700	0.30	411.82 Kb	1		Recessive	Recessive	no			NAD-dependent glycerol-3-phosphate dehydrogenase
SIS1	YNL007C	1.25±0.3	600	0.19	164.77 Kb	0		Recessive		no			Type II HSP40 co-chaperone that interacts with the HSP70 protein Ssa1p
BAP2	YBR068C	1.50±1	500	0.23	373.86 Kb	0		Recessive	Recessive	no	no		High-affinity leucine permease
RPL33B	YOR234C	1.55±0	1000	0.16	311.88 Kb			Recessive	Did not evolve	no			Ribosomal protein L37 of the large (60S) ribosomal subunit
HXT3	YDR345C	1.72±0.8	1300	0.17	367.26 Kb	1		Recessive	Recessive	no	no		Low affinity glucose transporter
PIL1	YGR086C	1.77±0.3	900	0.19	440.33 Kb	1	1	Two modes	Two modes	Yes (dominant colonies)		diploids (after evolution)	Primary component of eisosomes, which are large immobile cell cortex structures associated with endocytosis
ERG6	YML008C	2.20±0	400	0.25	251.84 Kb	-1		Recessive	Recessive	no	no		Delta(24)-sterol C-methyltransferase, converts zymosterol to fecosterol in the ergosterol biosynthetic pathway

a. See materials and methods for calculation
b. η=std (flourecence)/mean(flourecence)
c. 1=exist, -1= does not exist, o=not assigned
